# Species identification by MALDI-TOF MS and *gap* PCR–RFLP of non-*aureus Staphylococcus*, *Mammaliicoccus*, and *Streptococcus* spp. associated with sheep and goat mastitis

**DOI:** 10.1186/s13567-022-01102-4

**Published:** 2022-10-15

**Authors:** Nives Maria Rosa, Martina Penati, Sara Fusar-Poli, Maria Filippa Addis, Sebastiana Tola

**Affiliations:** 1 Istituto Zooprofilattico Sperimentale della Sardegna “G. Pegreffi”, Via Duca degli Abruzzi 8, 07100 Sassari, Italy; 2 Dipartimento di Medicina Veterinaria e Scienze Animali, Università degli Studi di Milano, Via dell’Università 6, 26900 Lodi, Italy; 3 MILab, Università degli Studi di Milano, Via dell’Università 6, 26900 Lodi, Italy

**Keywords:** Small ruminant, milk, coagulase-negative staphylococci, streptococci, mammaliicocci, NAS, NASM, species identification, *Gap* gene

## Abstract

Staphylococci and streptococci are common causes of intramammary infection in small ruminants, and reliable species identification is crucial for understanding epidemiology and impact on animal health and welfare. We applied MALDI-TOF MS and *gap* PCR–RFLP to 204 non-*aureus* staphylococci (NAS) and mammaliicocci (NASM) and to 57 streptococci isolated from the milk of sheep and goats with mastitis. The top identified NAS was *Staphylococcus epidermidis* (28.9%) followed by *Staph. chromogenes* (27.9%), *haemolyticus* (15.7%), *caprae*, and *simulans* (6.4% each), according to both methods (agreement rate, AR, 100%). By MALDI-TOF MS, 13.2% were *Staph. microti* (2.9%), *xylosus* (2.0%), *equorum*, *petrasii* and *warneri* (1.5% each), *Staph. sciuri* (now *Mammaliicoccus sciuri*, 1.0%), *arlettae*, *capitis*, *cohnii*, *lentus* (now *M. lentus*), *pseudintermedius*, *succinus* (0.5% each), and 3 isolates (1.5%) were not identified. PCR–RFLP showed 100% AR for *Staph. equorum*, *warneri*, *arlettae*, *capitis*, and *pseudintermedius*, 50% for *Staph. xylosus*, and 0% for the remaining NASM. The top identified streptococcus was *Streptococcus uberis* (89.5%), followed by *Strep. dysgalactiae* and *parauberis* (3.5% each) and by *Strep. gallolyticus* (1.8%) according to both methods (AR 100%). Only one isolate was identified as a different species by MALDI-TOF MS and PCR–RFLP. In conclusion, MALDI-TOF MS and PCR–RFLP showed a high level of agreement in the identification of the most prevalent NAS and streptococci causing small ruminant mastitis. Therefore, *gap* PCR–RFLP can represent a good identification alternative when MALDI-TOF MS is not available. Nevertheless, some issues remain for *Staph. haemolyticus,* minor NAS species including *Staph. microti*, and species of the novel genus *Mammaliicoccus*.

**Supplementary Information:**

The online version contains supplementary material available at 10.1186/s13567-022-01102-4.

## Introduction

Mastitis is one of the most common and costly diseases affecting dairy sheep and goats. Therefore, monitoring and maintaining udder health is critical for small ruminant welfare and dairy production yield and quality [1, 2]. Infectious mastitis outbreaks can be caused by a wide variety of bacterial species, including the genera *Staphylococcus* (*Staph.*) and *Streptococcus* (*Strep.*) [2, 3]. Among *Staphylococcus* spp., non-*aureus* staphylococci (NAS) are the most prevalent and cause mainly subclinical intramammary infection (IMI) in both ewes and goats, leading to significant economic losses and reduced animal welfare [4, 5]. Recently, based on 16S rRNA sequences, five NAS species were reclassified and assigned to the novel genus *Mammaliicoccus* with *Mammaliicoccus sciuri* as the type species [6]. These species are collectively indicated by the acronym NASM. The large NASM bacterial group includes numerous species with different prevalence and epidemiology [2, 3, 7]. From studies on bovine subclinical mastitis, it is clear that some species are more associated with IMI, subclinical mastitis and somatic cell count (SCC) increase than others, which are instead more related to the farm environment or the mammary gland microbiota [6, 8–10]. Obtaining precise information on the species of NASM causing IMI and mastitis is therefore crucial for understanding the epidemiology and the respective roles in mammary gland health and disease in order to make more appropriate management decisions when these bacteria are isolated from the milk of small ruminants [7, 11].

In the microbiological laboratory, staphylococci and streptococci isolated from milk samples are mainly identified at the species level by means of biochemical tests or commercial biochemical galleries. However, these methods often fail in correctly identifying bacterial species of veterinary interest, also because they have been optimized for the strains associated with human infections [12–15]. In the last decade, matrix-assisted laser desorption ionization time-of-flight mass spectrometry (MALDI-TOF MS) has emerged as a fast and accurate microbial identification approach [16] and is being successfully applied to bacteria isolated from bovine milk [17–19]. However, there are scarce reports of its performance in the identification of bacteria isolated from small ruminant milk, especially concerning staphylococci and sheep. Identification methods based on molecular rather than phenotypic characteristics can also provide a reliable alternative. Accordingly, the Istituto Zooprofilattico Sperimentale della Sardegna (IZSSA) has developed, comparatively assessed, and implemented a PCR-restriction fragment length polymorphism (PCR–RFLP) method based on PCR amplification of the *gap* gene followed by *Alu*I enzyme digestion for the identification of staphylococci and streptococci isolated from routine diagnosis [12, 13, 20].

In this study, we applied MALDI-TOF MS and *gap* PCR–RFLP for the species identification of staphylococci and streptococci isolated from the milk of sheep and goats with mastitis. Then, we compared their respective results to identify potential issues and solutions. Bacterial isolates were collected in Sardinia, the region with the largest small ruminant population in Italy.

## Materials and methods

### Bacterial isolates

From January 2021 to May 2022, 204 NASM and 57 *Streptococcus* spp. were isolated from sheep and goat milk samples that routinely arrive at the IZSSA laboratories for identification of the IMI agent. Milk samples are accompanied by a form with a checkbox indicating the presence of clinical mastitis/visible milk alterations to be selected by the farm veterinarian. Only isolates derived from samples with these characteristics were included in the study. Milk samples were cultured following the standard procedures provided by the IZSSA. Briefly, 10 µL of milk were seeded in 5% sheep blood agar, incubated at 37 °C, and evaluated at 24 and 48 h. Upon growth of more than one morphologically different bacterial colony, identifications were not performed, and the sample was classified as “mixed bacterial flora”. Colonies were re-isolated in blood agar and examined with Gram stain, catalase, and coagulase tests to discriminate between staphylococci and streptococci. All isolates were stored at −20 °C in Brain Heart Infusion broth (BHI, Beckton-Dickinson, Sparks, MD, USA) containing 20% glycerol, until further investigation. Only one NASM or streptococcus isolate was selected from each farm for identification by MALDI-TOF MS and PCR–RFLP, for a total of 261 non-duplicate isolates. A geographic map indicating the site of collection of each isolate in the different provinces of Sardinia was created with the software Microsoft Power BI using the farm code of each flock. The geo-referenced coordinates were extracted from the Banca Dati Nazionale (BDN) of the Italian Health Ministry. Additional file 1 illustrates the animal species originating the isolates and their geographical distribution.

### MALDI-TOF MS for bacterial identification

At the IZSSA, all bacterial isolates were retrieved from the frozen archives, seeded in 5% sheep blood agar, and incubated at 37 °C. All isolates were passaged twice in the solid medium before identification at 24 h of growth. For MALDI-TOF MS analysis, the direct colony transfer protocol was applied. A small amount of an isolated colony was deposited in duplicate wells of disposable target plates using a toothpick, overlaid with 1 µL of α-cyano-4-hydroxycinnamic acid (HCCA) solution in 50% acetonitrile, 47.5% water, 2.5% trifluoroacetic acid (Bruker Daltonik GmbH, Bremen, Germany), and left to dry. The target plates were prepared at the IZZSA in the afternoon, placed in an empty disposable target container once dry, and transported at room temperature to the animal infectious diseases laboratory at the University of Milan (MILab). In the morning of the following day, i.e. within 24 h as recommended by the MALDI Biotyper™ (MBT) Bruker user manual, the mass spectra were acquired with the MBT Microflex LT/SH MALDI-TOF mass spectrometer (Bruker Daltonik GmbH) in the positive mode. Each target plate included two spots of Bacterial Test Standard (Bruker Daltonik GmbH). The obtained spectra were interpreted against the MBT Compass® Library Revision H (2021), covering 3893 species/entries. The *Staphylococcus* and *Streptococcus* species included in this library revision are listed in Additional file 2. The two *Mammaliicoccus* species *M. sciuri and M. lentus* were still reported as *Staph. sciuri* and *Staph. lentus* in the library revision available at the time of this study. The following similarity log score thresholds were considered [16]: a log score ≥ 2.0 indicated a reliable species level identification, while a log score between 1.7 and 2.0 indicated a presumptive species level identification. Identifications with log scores below 1.7 were considered unreliable. All samples producing scores below 1.7 were processed again with the direct transfer, extended direct transfer, and protein extraction procedures. Specifically, for the extended direct transfer procedure, after depositing a small amount of the isolated colony in duplicate wells of the disposable target plate using a toothpick, the sample was overlaid with 1 µL of 70% formic acid and left to dry before adding the HCCA matrix solution. For the protein extraction procedure, bacteria from 4 isolated colonies were deposited into 300 µL of HPLC-grade water and vortexed. Then, 900 µL of ethanol absolute were added, the tube was vortexed again, and centrifuged for 2 min at maximum speed in a microcentrifuge. The supernatant was removed carefully, and the pellet was left to dry for 10 min at room temperature. Once dried, the pellet was thoroughly resuspended in 25 µL of 70% formic acid, 25 µL of acetonitrile were added, and the suspension was mixed by pipetting. The sample was centrifuged again as above, and 1 µL of supernatant was deposited on the MALDI target and left to dry before overlaying with the HCCA matrix.

### DNA extraction and PCR amplification

At the IZSSA, genomic DNA was extracted from all 261 isolates and Type/Reference Strains (T/RS) as described previously [12]. Species identification was based on PCR amplification of the glyceraldehyde-3-phosphate dehydrogenase gene (*gap*) gene [20, 21]. The primers GF-1 (5’-ATGGTTTTGGTAGAATTGGTCGTTTA-3’) and GR-2 (5’-GACATTTCGTTATCATACCAAGCTG-3’) were used for staphylococci whereas the primers Strept-gap-F (5’-ACTCAAGTGTACGAACAAGT-3’) and Strept-gap-R (5’-GTCTTGCATTCCGTCGTAT-3’) for streptococci. PCR was performed in a reaction mixture containing 2.5 µL 10 × reaction buffer, 1.5 µL dNTPs 1.25 mM, 1 µL of each primer (25 pmol each), 1 µL DNA template, 0.5 µL Fast Taq (Roche, Basel, Switzerland), and distilled water up to 25 µL. Reactions were carried out in an automated DNA thermal cycler (GeneAmp 9700, Applied Biosystems, CA, USA). Amplification conditions: initial denaturation for 5 min at 95 °C followed by 30 cycles of 1 min at 95 °C, 1 min at 50 °C (for staphylococci)/1 min at 54 °C (for streptococci), and 1 min at 72 °C with a final extension step of 10 min at 72 °C. The 933-bp (staphylococci) and 945-bp (streptococci) amplicons were examined by electrophoresis in 1% agarose gels, stained with Sybr^®^ Safe DNA gel stain (Invitrogen, CA, USA), and visualized under a UV transilluminator.

### Restriction fragment length polymorphism (RFLP) analysis

Fifteen microliters of both PCR amplifications were digested in a 30 µL volume containing 10 × FastDigest Green buffer, 0.25 µL of 20 mg/mL acetylated BSA, and 1 µL of FastDigest *Alu*I enzyme (Thermo Scientific, CA, USA). Reaction mixtures were incubated at 37 °C for 15 min and directly loaded on the precast gels. Twenty microliters of digested *gap* amplifications were loaded in 12% Bis–Tris NuPAGE^™^ gels (Invitrogen) and then electrophoresed in a vertical gel apparatus. For staphylococci, the following 14 T/RS were used as reference strains for restriction pattern comparison: *Staph. epidermidis* ATCC 35983, *Staph. xylosus* ATCC 29971^ T^, *Staph. saprophyticus* ATCC 15305^ T^, *Staph. capitis* ATCC 27840^ T^, *Staph. haemolyticus* ATCC 29970^ T^, *Staph. simulans* ATCC 27848^ T^, *Staph. warneri* ATCC 27836 T, *Staph. arlettae* ATCC 43957^ T^, *Staph. chromogenes* ATCC 43764^ T^, *Staph. equorum* ATCC 43958^ T^, *Staph. caprae* ATCC 35538^ T^, *Staph. sciuri* ATCC 29062^ T^, *Staph. hiycus* ATCC 11249 ^T^, and *Staph. intermedius* ATCC 29663^ T^. The *gap* PCR–RFLP patterns used for *Staphylococcus* species assignment are illustrated in Additional file 3. For streptococci, the following 7 T/RS were used as reference strains for restriction pattern comparison: *Strep. uberis* ATCC 700407, *Strep. dysgalactiae* subsp. *dysgalactiae* ATCC 43078^ T^, *Strep. dysgalactiae* subsp. *equisimilis* DSM 23147^ T^, *Strep. agalactiae* ATCC 13813^ T^, *Strep. gallolyticus* subsp. *gallolyticus* ATCC 49475, *Strep. equi* subsp. *zooepidermicus* NCTC 6180, and *Strep. suis* ATCC 43765. The *gap* PCR–RFLP patterns used for *Streptococcus* species assignment are illustrated in Additional file 4. All reference strain species identities were confirmed by MALDI-TOF MS.

### Amplicon sequencing

The *gap* gene amplicons of all isolates with PCR–RFLP profiles different from those of *Staphylococcus* and *Streptococcus* T/RS were sequenced at BMR Genomics with the Sanger sequencing option. The nucleotide sequences were compared to sequences in the GenBank database using the Basic Local Alignment Search Tool (BLAST).

### Comparison of PCR–RFLP and MALDI-TOF MS results

All the data related to MALDI-TOF MS results, log scores, PCR–RFLP identification, *gap* gene sequencing results, and animal species, were plotted with Microsoft Excel for calculation of agreement ratio (AR), score distribution, and relative percentage values. Plots were generated with Microsoft Excel.

## Results

### *Isolates* and geographical distribution.

From January 2021 to May 2022, 204 NASM and 57 streptococci, for a total of 261 isolates, were obtained from sheep and goat milk samples sent to the IZSSA for microbiological analysis with a diagnosis of clinical mastitis/visible milk alterations by the farm veterinarian. Ovine isolates were 246, of which 191 NASM and 55 streptococci. Caprine isolates were 15, of which 13 NASM and 2 streptococci (Additional file 5).

### MALDI-TOF MS of NASM isolates

All 204 isolates typed as NASM by culture and primary biochemical tests were subjected to MALDI-TOF MS identification by processing two spots per colony with the direct transfer procedure after 24 h of bacterial growth. This enabled the successful species identification of 201 isolates (98.5%), with log scores ≥ 2.00 in 165 (80.9%) and between 1.70–1.99 in 36 (17.6%). For the remaining 3 isolates (1.5%), log scores were < 1.70 with no identification possible even after a second round of identification with the direct transfer, extended direct transfer, and protein extraction procedures (Table 1).

**Table 1:** **Summary of species identification results obtained by MALDI-TOF MS and** ***gap*** **PCR–RFLP on the 261 isolates evaluated in this study**

Species ID by MALDI-TOF MS^a^	N. of isolates^b^	Log score			AR with *gap* PCR-RFLP^f^
		≥ 2.0^c^	1.7–1.99^d^	< 1.7 (no ID)^e^	
Staphylococci					
*Staphylococcus epidermidis*	59 (28.9%)	55 (93.2%)	4 (6.8%)	–	59 (100%)^g^
*Staphylococcus chromogenes*	57 (27.9%)	53 (93.0%)	4 (7.0%)	–	57 (100%)^g^
*Staphylococcus haemolyticus*	32 (15.7%)	18 (56.3%)	14 (43.7%)	–	32 (100%)^g^
*Staphylococcus caprae*	13 (6.4%)	10 (76.9%)	3 (23.1%)	–	13 (100%)^g^
*Staphylococcus simulans*	13 (6.4%)	13 (100%)	–	–	13 (100%)^g^
*Staphylococcus microti*	6 (2.9%)	3 (50%)	3 (50%)	–	0 (0%)
*Staphylococcus xylosus*	4 (2.0%)^8^	2 (50%)	2 (50%)	–	2 (50%)^g^
*Staphylococcus equorum*	3 (1.5%)	3 (100%)	–	–	3 (100%)^g^
*Staphylococcus petrasii*	3 (1.5%)	3 (100%)	–	–	0 (0%)
*Staphylococcus warneri*	3 (1.5%)	2 (66.7%)	1 (33.3%)	–	3 (100%)^g^
*Staphylococcus sciuri*^*h*^	2 (1.0%)	2 (100%)	–	–	0 (0%)^g^
*Staphylococcus arlettae*	1 (0.5%)	–	1 (100%)	–	1 (100%)^g^
*Staphylococcus capitis*	1 (0.5%)	1 (100%)	–	–	1 (100%)^g^
*Staphylococcus cohnii*	1 (0.5%)	–	1 (100%)		0 (0%)
*Staphylococcus lentus*^*h*^	1 (0.5%)^9^	–	1 (100%)	–	0 (0%)
*Staphylococcus pseudintermedius*	1 (0.5%)	–	1 (100%)	–	1 (100%)^g^
*Staphylococcus succinus*	1 (0.5%)	–	1 (100%)	–	0 (0%)
Unidentified	3 (1.5%)	–	–	3 (100%)	–
Total staphylococci	204	165 (80.9%)	36 (17.6%)	3 (1.5%)	185 (90.7%)
Streptococci					
*Streptococcus uberis*	51 (89.5%)	48 (94.1%)	3 (5.9%)	–	51 (100%)^g^
*Streptococcus dysgalactiae*^910^	2 (3.5%)	2 (100%)	–	–	2 (100%)^g^
*Streptococcus parauberis*	2 (3.5%)	2 (100%)	–	–	2 (100%)^k^
*Streptococcus gallolyticus*	1 (1.8%)	1 (100%)	–	–	1 (100%)^g^
*Streptococcus suis*	1 (1.8%)	–	1 (100%)	–	0 (0%)^g^
Unidentified	0 (1.8%)	–	–	–	–
Total streptococci	57	53 (93.0%)	4 (7.0%)	0 (0%)	56 (98.2%)

^a^Based on the Bruker MALDI Biotyper System Compass® Library Revision H (2021), covering 3893 species/entries.

^b^Percent data represent the proportion of a given species isolated among all staphylococci or streptococci (within column).

^c^Percent data represent the proportion of isolates identified with MALDI log scores ≥ 2.0 among all isolates of the same species.

^d^Percent data represent the proportion of isolates identified with MALDI log scores 1.7–1.99 among all isolates of the same species.

^e^Percent data represent the proportion of isolates with MALDI log scores < 1.7 among all isolates of the same species and no identification possible (no ID).

^f^Number of isolates identified as the same species by MALDI-TOF MS and *gap* PCR–RFLP. Percent data represent the proportion of isolates identified as the same species (Agreement Rate, AR).

^g^The reference strain for this species was included in the *gap* PCR–RFLP identification panel (please see the materials and methods section for the reference isolate list).

^h^These species have been reclassified in the *Mammaliicoccus* genus [6].

^i^For one isolate, the first round of MALDI-TOF MS identification was not successful and it was repeated.

^j^*Streptococcus dysgalactiae* and *Streptococcus canis* cannot be resolved by MALDI-TOF MS.

^k^Both assigned by *gap* gene amplicon sequencing and alignment with sequences in the GenBank database using the Basic Local Alignment Search Tool (BLAST). Details are reported in Additional file 7.

The most frequently identified species were *Staph. epidermidis* (59, 28.9%), *Staph. chromogenes* (57, 27.9%), *Staph. haemolyticus* (32, 15.7%), *Staph. caprae* (13, 6.4%), and *Staph. simulans* (13, 6.4%), accounting for 85.3% of all NASM isolates. The remaining 13.2% was represented by *Staph. microti* (6, 2.9%), *Staph. xylosus* (4, 2.0%), *Staph. equorum*, *petrasii, and warneri* (3 each, 1.5%), *Staph. sciuri* (now *M. sciuri*) (2, 1.0%), and *Staph. arlettae*, *capitis*, *cohnii, lentus* (now *M. lentus*), *pseudintermedius*, and *succinus* (1 each, 0.5%).

### MALDI-TOF MS of streptococcus isolates

MALDI-TOF MS identification of the 57 streptococcus isolates included in this study enabled the successful species identification of all isolates (100%) with log scores ≥ 2.00 in 53 (93.0%) and between 1.7–1.99 in 4 (7.0%). The most frequently identified species was *Strep. uberis* (51, 89.5%), followed by *Strep. dysgalactiae* and *parauberis* (2 each, 3.5%), and by *Strep. gallolyticus* and *suis* (1 each, 1.8%) (Table 1).

### PCR–RFLP of NASM isolates

All 204 isolates typed as NASM by culture and primary biochemical tests were also subjected to species identification by *gap* PCR–RFLP. A total of 187 (91.7%) isolates showed restriction profiles identical to the reference strains, while 17 (8.3%) isolates showed a different PCR–RFLP pattern (Table 1). Upon *gap* gene sequencing (Additional file 6), 12 of them were identified as *Staph. chromogenes* (1), *devriesei* (1), *epidermidis* (1), *haemolyticus* (3) *hyicus* (1), *jettensis* (1), *muscae* (1), *pseudintermedius* (1), *pseudoxylosus* (1), and *simulans* (1), while 5 remaining isolates were classified as *Staph. muscae* (4) and *Staph. devriesei* (1) by matching with the isolate classified by *gap* gene sequencing (Table 2)*.*

**Table 2:** **Detail of the 20 discordant species identification results between MALDI-TOF MS and** ***gap*** **PCR–RFLP integrated with** ***gap*** **gene sequencing**

MALDI-TOF MS^a^	Log score	ID by *gap* PCR-RFLP^b^	ID by *gap* sequencing^c^ (% identity)^d^
Staphylococci			
*Staphylococcus microti*	1.99	–	*Staphylococcus muscae* (92.79%)
*Staphylococcus microti*	2.05	–	*Staphylococcus muscae*^*e*^
*Staphylococcus microti*	2.06	–	*Staphylococcus muscae*^*e*^
*Staphylococcus microti*	1.91	–	*Staphylococcus muscae*^*e*^
*Staphylococcus microti*	1.76	–	*Staphylococcus muscae*^*e*^
*Staphylococcus microti*	2.04	–	*Staphylococcus simulans* (99.89%)
*Staphylococcus petrasii*	2.1	*Staphylococcus haemolyticus*	–
*Staphylococcus petrasii*	2.03	*Staphylococcus haemolyticus*	–
*Staphylococcus petrasii*	2.24	–	*Staphylococcus jettensis* (99.29%)
*Staphylococcus sciuri*^*f*^	2.23	–	*Staphylococcus chromogenes* (98.75%)
*Staphylococcus sciuri*^*f*^	2.16	–	*Staphylococcus hyicus* (98.69%)
*Staphylococcus xylosus*	1.88	–	*Staphylococcus devriesei*^g^
*Staphylococcus xylosus*	1.81^ g^	–	*Staphylococcus pseudoxylosus* (99.78%)
*Staphylococcus cohnii*	1.7^ h^	–	*Staphylococcus haemolyticus* (98.91%)
*Staphylococcus lentus*^*f*^	1.8^ h^	–	*Staphylococcus epidermidis* (98.46%)
*Staphylococcus succinus*	1.93	*Staphylococcus epidermidis*	–
Unidentified	1.6	–	*Staphylococcus haemolyticus* (99.07%)
Unidentified	1.28	–	*Staphylococcus haemolyticus* (98.26%)
Unidentified	1.44	–	*Staphylococcus devriesei* (99.77%)
Streptococci			
*Streptococcus suis*	1.78	–	*Streptococcus ruminantium* (98.15%)

^a^Based on the Bruker MALDI Biotyper System Compass® Library Revision H (2021), covering 3893 species/entries.

^b^Species identification was assigned by matching the enzyme digestion pattern of the *gap* gene amplicon with the reference strain.

^c^All amplicons producing an enzyme digestion pattern different than the reference strains were subjected to genomic sequencing for species identification.

^d^Percent identity of the *gap* gene amplicon with sequences in the GenBank database using the Basic Local Alignment Search Tool (BLAST). Details are reported in Additional files 6 and 7.

^e^Based on the restriction pattern of the *Staphylococcus muscae* isolate identified by gap sequencing.

^f^These species have been reclassified in the *Mammaliicoccus* genus [6].

^g^Based on the restriction pattern of the *Staphylococcus devriesei* isolate identified by gap sequencing.

^h^The first round of MALDI-TOF MS identification was not successful and it was repeated.

As a result, the top three identified species were *Staph. epidermidis* (61, 29.9%), *Staph. chromogenes* (58, 28.4%), and *Staph. haemolyticus* (37, 18.1%), followed by *Staph. simulans* (14, 6.9%) *Staph. caprae* (13, 6.4%), *Staph. muscae* (5, 2.5%), *Staph. equorum* and *warneri* (3, 1.5%), *Staph. devriesei* and *xylosus* (2 each, 1.0%), and *Staph. arlettae*, *capitis*, *hyicus*, *jettensis*, *pseudintermedius*, and *pseudoxylosus* (1 each, 0.5%).

### PCR–RFLP of streptococcus isolates

Out of the 57 isolates typed as *Streptococcus* sp. by culture and primary biochemical tests, 53 (93.0%) showed *gap* PCR–RFLP profiles identical to the reference strains, while 4 (7.0%) did not. Upon *gap* gene sequencing (Additional file 7), these were identified as *Strep. parauberis* (2), *Strep. uberis* (1), and *Strep. ruminantium* (1) (Table 1 and Table 2). As a result, the top identified species was *Strep. uberis* (51, 89.5%), followed by *Strep. dysgalactiae* and *parauberis* (2 each, 3.5%), and by *Strep. gallolyticus* and *ruminantium* (1 each, 1.8%).

### Comparison of gap PCR–RFLP and MALDI-TOF MS results

**Figure 1:**
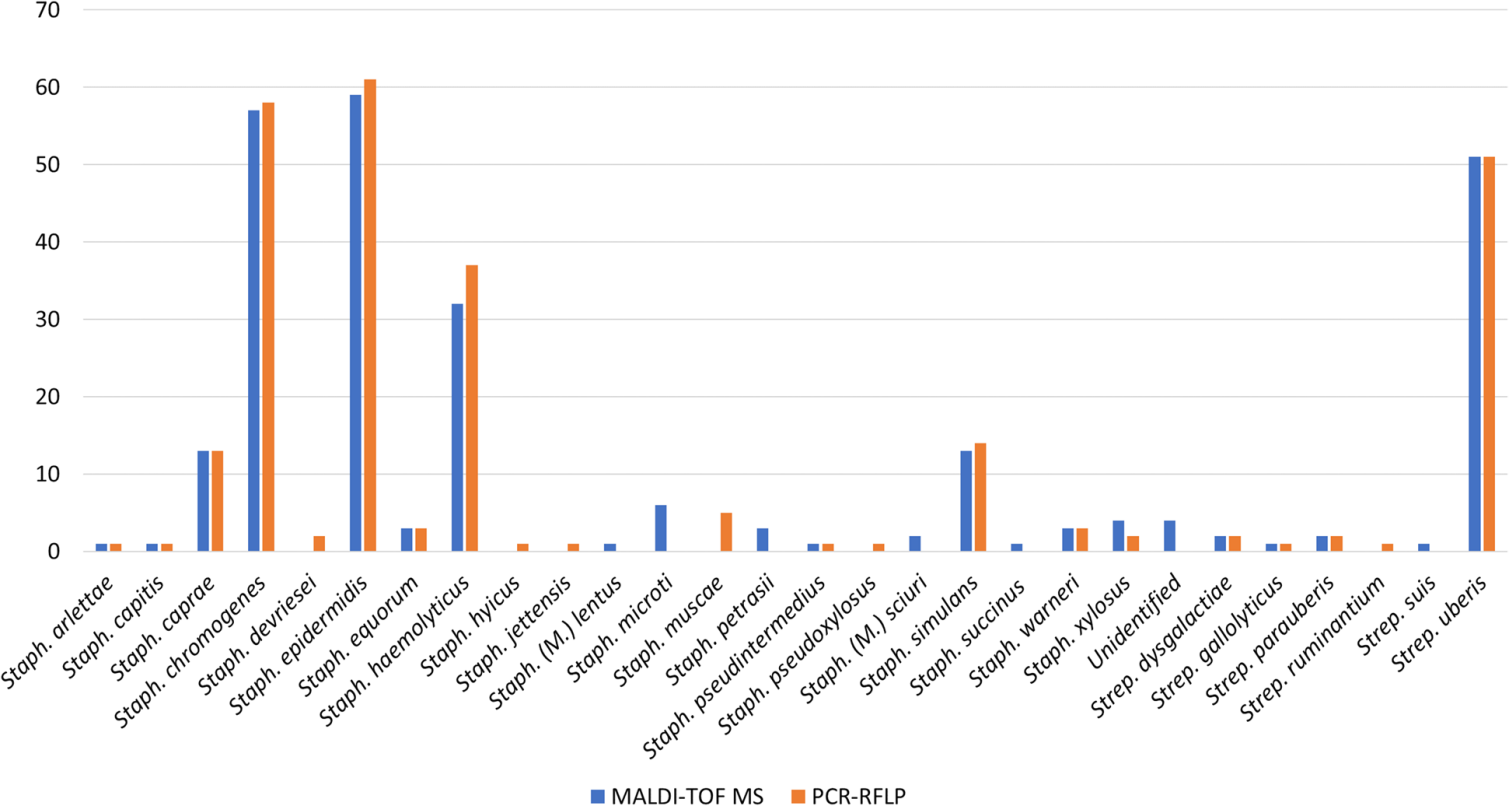
**Histogram of the species distribution**. The graph illustrates the number of *Staphylococcus* (*Mammaliicoccus*) and *Streptococcus* spp. identified in this study by MALDI-TOF MS (orange) and *gap* PCR–RFLP (blue).

The species identification results obtained by MALDI-TOF MS and *gap* PCR–RFLP were in general agreement as detailed in Table 1 and illustrated in Figure 1.

For the 5 most frequently identified NAS (*Staph. epidermidis*, *chromogenes*, *haemolyticus*, *caprae*, and simulans) the agreement rate (AR) between MALDI-TOF MS and *gap* PCR–RFLP was 100%. Log scores were mostly ≥ 2.00, although for *Staph. haemolyticus* only 56.3% of the identifications had log scores ≥ 2.00. The AR between the two identification methods was 100% also for *Staph. equorum*, *warneri*, *arlettae*, *capitis*, and *pseudintermedius*. On the other hand, AR was 50% for *Staph. xylosus* and 0% for *Staph. microti*, *Staph. petrasii*, *Staph sciuri* (now *M. sciuri)*, *Staph. cohnii*, *Staph. lentus* (now *M. lentus*), and *Staph. succinus* (Table 1).

The discordant species identifications between the two approaches are reported in Table 2. Of note was the case of 6 isolates identified as *Staph. microti* by MALDI-TOF MS and as *Staph. muscae* (5 isolates) and *Staph. simulans* (1 isolate) by *gap* sequencing. Among less frequently identified NASM species, only *gap* sequencing provided as identifications *Staph. muscae*, *devriesei*, *hyicus*, *jettensis*, and *pseudoxylosus*, while only MALDI-TOF MS provided *Staph. microti, Staph. petrasii, Staph. sciuri* (now *M. sciuri)*, *Staph. lentus* (now *M. lentus),* and *Staph. succinus*.

For streptococci, the AR between MALDI-TOF MS and *gap* PCR–RFLP was 98.2% (56 of 57). The AR was 100% for the identification of *Strep. uberis*, with log scores ≥ 2.00 in 94.1% of cases. The AR was also 100% for *Strep. dysgalactiae*, *parauberis*, and *gallolyticus*. Only one species was identified as *Strep suis* by MALDI-TOF MS (log score 1.78) and as *Strep. ruminantium* by *gap* sequencing (Table 2).

## Discussion

NAS and streptococci are the most prevalent etiological agents of small ruminant mastitis [2, 3]. However, NAS, mammaliicocci, and streptococci can also be found in milk as contaminants, as well as components of the mammary gland microbiota [6, 9, 10, 22]. Obtaining a reliable species identification is therefore essential for understanding their epidemiology and roles in mammary gland health and disease in order to make more meaningful management decisions [7, 8, 23].

In the last decade, MALDI-TOF MS has emerged as a dependable method for microbial identification and is being increasingly applied to bacteria and fungi isolated from bovine milk. When MALDI-TOF MS instrumentation is available in the laboratory, this approach is rapid, cost-effective, high-throughput, reliable, and does not require specific knowledge of mass spectrometry or molecular biology. An isolated colony is sufficient for identification and results are available within minutes. Thanks to the short analytical times, a high number of targets can be processed during the day, making it possible to identify thousands of microbial isolates. A further advantage of MALDI-TOF MS is the possibility of creating personalized spectrum libraries including isolates of specific interest, thereby improving bacterial identification reliability [24].

In the last decade, the IZSSA has developed and applied in its diagnostic routine a *gap* PCR–RFLP method enabling the identification of NAS and streptococci isolated from small ruminant milk [12, 13, 20]. This approach is reliable but moderately expensive in terms of materials and labor as it requires DNA extraction, PCR amplification, amplicon digestion, electrophoresis, and result interpretation by comparison with RFLP profiles from reference strains. The restricted number of reference strains is also a limitation, and it may eventually require *gap* gene amplicon sequencing for a tentative identification by homology. However, *gap* PCR–RFLP may represent a valuable alternative when MALDI-TOF instrumentation is not available. In fact, in spite of the low cost per test, the mass spectrometer acquisition costs are still too high for many veterinary diagnostic facilities, especially in low-resource contexts or in peripheral laboratories. As a further consideration, scarce data are available in the literature on the application of MALDI-TOF MS identification to the microorganisms isolated from small ruminant milk, especially concerning NAS and sheep.

In this work, we carried out the identification of NASM and streptococci isolated from the milk of small ruminants with mastitis by MALDI-TOF MS and *gap* PCR–RFLP integrated with amplicon sequencing. As a result, the species most frequently identified in our study were in line with those reported as causing small ruminant IMI worldwide [11, 25], that is *Staph. epidermidis*, *chromogenes*, *haemolyticus*, *caprae* and *simulans* for NAS, and *Strep. uberis* for streptococci. Notably, the two identification approaches showed a very high level of agreement, even for MALDI-TOF MS identifications with log scores ≥ 1.7. A cutoff log score ≥ 1.7 for species identification has been validated by various authors [19, 26, 27] as highly appropriate and accurate for bovine NAS. Han et al. [27] found that the ≥ 1.7 log score threshold enabled to reach a significantly higher level of NAS species identification without sacrificing specificity. Cameron et al. [28] also found that the reduction of the species level cutoff improved method performance from 64 to 92% when classifying bovine-associated NAS isolates. In the more recent study by Conesa et al. [19] the ≥ 1.7 score made it possible to successfully identify 36 more strains, as validated by the comparison with genotypic methods. Based on our results, a log score threshold of ≥ 1.7 could be considered reliable also for the most prevalent small ruminant NAS.

For *Staph haemolyticus*, however, the log scores were generally lower, as 43.7% of isolates had values < 2.0. Notably, 2 isolates were identified as *Staph. haemolyticus* by PCR–RFLP and as *Staph. petrasii* by MALDI-TOF MS with log scores > 2.0. Moreover, the *gap* gene of three isolates showed a very high sequence identity to *Staph. haemolyticus* but these were either identified as *Staph. cohnii* with a very low log score (1.7) or were not identified by MALDI-TOF MS. In our routine work, *Staph. haemolyticus* isolated from cow milk is also typically identified with lower scores among the most prevalent *Staphylococcus* spp. (M.F.A., personal communication). *Staph. haemolyticus* may be more problematic to identify because of a higher genomic variability and similarity of marker genes with other species. Wanecka et al. [14] found that 27 of 33 of their *Staph. haemolyticus* isolates had 99.5–100% similarity of the 16S rRNA gene with *Staph. petrasii* subsp. *jettensis* (now *Staph. petrasii* subsp. *petrasii*), *Staph. hominis*, *Staph. epidermidis*, or *Staph. devriesei*. Also recently, some *Staph. haemolyticus* have been reclassified as *Staph. borealis* [29]. Therefore, molecular techniques may have limitations including insufficient discriminatory power in the case of closely related species or the lack of quality of sequences deposited in the GenBank [30], as also observed in this work for *gap* gene sequencing. On the other hand, the absence of reference spectra for these highly similar minor species in the MALDI-TOF MS database might be the reason for their identification as *Staph. haemolyticus* with lower log scores.

All the 6 isolates identified as *Staph. microti* by MALDI-TOF MS could not be identified by *gap* PCR–RFLP. The *gap* gene sequence showed the highest identity with *Staph. muscae* (5 out of 6) and *Staph. simulans* (1 out of 6). *Staph. microti* has been described for the first time by Novàkovà et al. in 2010 [31] as an isolate from *Microtus arvalis*, the common vole, with *Staph. muscae* as the nearest relative. Its report as a staphylococcal species associated with mastitis in bovine and bubaline cows is increasing in association with the implementation of MALDI-TOF MS for NAS identification [18, 32, 33]. However, in consideration of the high similarity between these species, as well as with other closely related species not included in the Bruker spectrum library such as *Staph. rostri*, this will require further evaluation, possibly followed by spectrum library integration.

An isolate identified as *Staph. petrasii* by MALDI-TOF MS with log score ≥ 2.00 was identified as *Staph. jettensis* upon *gap* gene sequencing. The species description of *Staph. petrasii* has been emended, and *Staph. jettensis* should be reclassified as a novel subspecies within *Staph. petrasii* for which the name *Staphylococcus petrasii* subsp. *jettensis* subsp. nov [34]. As discussed above, the identification of this NAS as *Staph. jettensis* by *gap* gene sequencing may have resulted from matching with GenBank sequences that were not updated following taxonomic reclassification.

In 2020, five NAS species were proposed to be reassigned to the novel *Mammaliicoccus* genus, including *Staph. sciuri* and *Staph. lentus*, with *M. sciuri* as the type species [6]. The same authors proposed the reclassification of *Staph*. *cohnii* *subsp*. urealyticus as the novel species *Staph. urealyticus*. The *Mammaliicoccus* genus is not included in the current release of the Bruker library. Therefore, this should be considered when these species are identified by MALDI-TOF MS with the MBT System and the commercial spectrum library release available at the time of this study.

Concerning the two isolates identified as *Staph. devriesei* by *gap* gene sequencing and PCR–RFLP, one was not identified by MALDI-TOF MS, while the other was identified as *Staph. xylosus* with a score < 2.00. Moreover, one isolate identified as *Staph. hyicus* by *gap* gene sequencing was identified as *Staph. sciuri* by MALDI-TOF MS with score ≥ 2.00. *Staph. devriesei* falls in the *Staph. haemolyticus* group [11], and *Staph. sciuri* is also reported as belonging to a separate group than *Staph. hyicus*. Nevertheless, *Staph. hyicus* can be difficult to differentiate from *Staph. agnetis* [35] and the latter species was not present in the MALDI-TOF MS spectrum database.

An isolate was identified as *Staph. xylosus* by MALDI-TOF MS after a second round of identification with score 1.81. The same isolate was identified as *Staph. pseudoxylosus* by gap gene sequencing; however, this species was not included in the spectrum library. Analogously, no *Strep. ruminantium* spectra were present. However, for this latter species a presumptive identification as *Strep. suis* was provided, with score ≥ 1.7. *Strep. ruminantium* is indeed a new species of the *suis* group. *Strep. suis* includes 35 serotypes, of which 6 have been re-classified to other bacterial species [36]. Among them, *Strep. suis* serotype 33 has been recently classified as a new species, *Streptococcus ruminantium* [37], of which the reference strain was originally isolated from a lamb. As the two species are biochemically very similar, differentiation is difficult and a PCR specific for *Strep. ruminantium* has recently been described [38]. All other streptococci showed a very high agreement rate; therefore, both MALDI-TOF MS and PCR–RFLP enable to overcome the known difficulties in the identification of Gram-positive, catalase-negative cocci by biochemical reactions [39, 40] also with streptococci isolated from small ruminant milk.

As a further consideration, in this work we carried out microbial cultivation and target preparation at the IZSSA in Sassari, Sardinia, and MALDI-TOF MS identification at the University of Milan in Lodi, Lombardy, on the following day. According to the Bruker MBT User Manual, result reliability is maintained if spectra are generated within 24 h from target preparation. Therefore, this opens the possibility that these may be prepared in one laboratory and sent to a shared core facility for identification within the following day, provided that convenient logistic solutions are in place. This approach would enable to contain instrumental costs while centralizing the mass spectrometry instrumentation and the spectrum library, and it might also represent a reasonable alternative to the *gap* gene sequencing to integrate the PCR–RFLP identification approach. If planning such a setup, however, it would be advisable to thoroughly assess the impact of target transportation temperatures and conditions on reliability of the MALDI-TOF MS identification results.

In conclusion, our results concerning the species of NASM and streptococci associated with sheep and goat mastitis were in line with those reported as causing small ruminant IMI worldwide [11, 25]. For the most prevalent species, MALDI-TOF MS and *gap* PCR–RFLP provided comparable results. Therefore, *gap* PCR–RFLP can offer a reliable identification alternative when MALDI-TOF MS is not available, but restriction profiles differing from the validated reference isolates [12, 13, 20] may not be easily resolved by *gap* gene sequencing. Concerning MALDI-TOF MS, integrating the spectrum library with small ruminant strains of *Staph. haemolyticus* as well as of *Staph. microti* and their closely related species might further improve identification performances and it is advised.

## Supplementary Information

**Additional file 1: Geographical distribution of all 261 isolates included in this study.**

**Additional file 2:** ***Staphylococcus*** **and** ***Streptococcus*** **species included in the MBT Compass® Library Revision H (2021).**

**Additional file 3:** **Restriction fragment length polymorphism (RFLP) pattern of PCR products of the** ***gap*** **gene obtained after digestion with** ***Alu*I and used for** ***Staphylococcus*** **species assignment.**

**Additional file 4:** **Restriction fragment length polymorphism (RFLP) pattern of PCR products of the** ***gap*** **gene obtained after digestion with** ***Alu*I and used for** ***Streptococcus*** **species assignment.**

**Additional file 5:** **Excel file detailing PCR–RFLP identification,** ***gap*** **gene sequencing information where obtained, MALDI-TOF MS identification, best log score, and animal species.**

**Additional file 6:** **Genomic sequence of the** ***gap*** **gene and sequence similarity data for staphylococci.**

**Additional file 7:** **Genomic sequence of the** ***gap*** **gene and sequence similarity data for streptococci.**
