# Supplementary material for: Species identification by MALDI-TOF MS and gap PCR–RFLP of non-aureus Staphylococcus, Mammaliicoccus, and Streptococcus spp. associated with sheep and goat mastitis
Source: Vet Res. 2022 Oct 15;53:84. doi: 10.1186/s13567-022-01102-4 (PMC9569034; doi:10.1186/s13567-022-01102-4)
Supplement: Supplementary file 1 — Additional file 1: Geographical distribution of all 261 isolates included in this study. [file 13567_2022_1102_MOESM1_ESM.docx]

**Additional file 1**. **Geographical distribution of all 261 isolates included in this study.** The map reports the location of the 204 non-*aureus Staphylococcus* and *Mammaliicoccus* (NASM), and 57 *Streptococcus* isolates in Sardinia, Italy. Each point represents an individual isolate. Circles indicate NASM: red for sheep milk, and yellow for goat milk. Triangles indicate streptococci: blue for sheep milk, and fuchsia for goat milk.

**
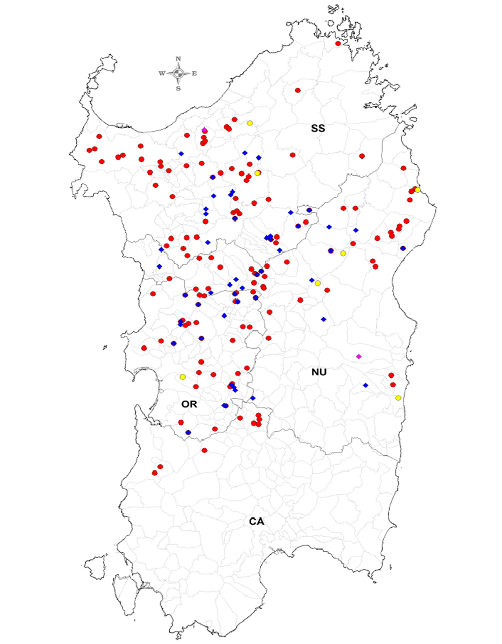
**
