# Supplementary material for: Species identification by MALDI-TOF MS and gap PCR–RFLP of non-aureus Staphylococcus, Mammaliicoccus, and Streptococcus spp. associated with sheep and goat mastitis
Source: Vet Res. 2022 Oct 15;53:84. doi: 10.1186/s13567-022-01102-4 (PMC9569034; doi:10.1186/s13567-022-01102-4)
Supplement: Supplementary file 2 — Additional file 2: Staphylococcus and Streptococcus species included in the MBT Compass® Library Revision H (2021). [file 13567_2022_1102_MOESM2_ESM.pdf]

Additional File 2. *Staphylococcus* and *Streptococcus* species included in the MBT Compass Library Rev. H (2021), Document Revision E, covering 3893 species/entries.

***Staphylococcus* spp.**	***Streptococcus* spp.**
Staphylococcus argenteus	*Streptococcus acidominimus*
Staphylococcus arlettae	*Streptococcus agalactiae*
Staphylococcus aureus	*Streptococcus alactolyticus*
Staphylococcus auricularis	*Streptococcus anginosus*
Staphylococcus borealis	*Streptococcus australis*
Staphylococcus capitis	*Streptococcus caballi*
Staphylococcus caprae	*Streptococcus canis*
Staphylococcus carnosus	*Streptococcus castoreus*
Staphylococcus chromogenes	*Streptococcus constellatus*
Staphylococcus cohnii	*Streptococcus criceti*
Staphylococcus condimenti	*Streptococcus cristatus*
Staphylococcus delphini	*Streptococcus dentirousetti*
Staphylococcus devriesei	*Streptococcus devriesei*
Staphylococcus epidermidis	*Streptococcus didelphis*
Staphylococcus equorum	*Streptococcus downei*
Staphylococcus felis	*Streptococcus dysgalactiae*
Staphylococcus fleurettii	*Streptococcus entericus*
Staphylococcus gallinarum	*Streptococcus equi*_ssp_*equi*
Staphylococcus haemolyticus	*Streptococcus equi*_ssp_*ruminatorum*
Staphylococcus hominis	*Streptococcus equi*_ssp_*zooepidemicus*
Staphylococcus hyicus	*Streptococcus equinus*
Staphylococcus intermedius	*Streptococcus ferus*
Staphylococcus kloosii	*Streptococcus gallinaceus*
Staphylococcus lentus	*Streptococcus gallolyticus*
Staphylococcus lugdunensis	*Streptococcus gordonii*
Staphylococcus lutrae	*Streptococcus halichoeri*
Staphylococcus massiliensis	*Streptococcus henryi*
Staphylococcus microti	*Streptococcus hyointestinalis*
Staphylococcus muscae	*Streptococcus hyovaginalis*
Staphylococcus nepalensis	*Streptococcus infantarius*
Staphylococcus pasteuri	*Streptococcus infantis*
Staphylococcus petrasii	*Streptococcus iniae*
Staphylococcus pettenkoferi	*Streptococcus intermedius*
Staphylococcus piscifermentans	*Streptococcus lutetiensis*
Staphylococcus pseudintermedius	*Streptococcus macacae*
Staphylococcus saccharolyticus	*Streptococcus marimammalium*
Staphylococcus saprophyticus	*Streptococcus massiliensis*
Staphylococcus schleiferi	*Streptococcus merionis*
Staphylococcus schweitzeri	*Streptococcus minor*
Staphylococcus sciuri	*Streptococcus mitis*
Staphylococcus simiae	*Streptococcus moroccensis*
Staphylococcus simulans	*Streptococcus mutans*
Staphylococcus sp[2]	*Streptococcus oralis*
Staphylococcus stepanovicii	*Streptococcus orisratti*
Staphylococcus succinus	*Streptococcus orisuis*
Staphylococcus vitulinus	*Streptococcus ovis*
Staphylococcus warneri	*Streptococcus parasanguinis*
Staphylococcus xylosus	*Streptococcus parauberis*
	Streptococcus penaeicida
	Streptococcus peroris
	Streptococcus phocae
	Streptococcus pluranimalium
	Streptococcus plurextorum
	Streptococcus pneumoniae
	Streptococcus porci
	Streptococcus porcinus
	Streptococcus porcorum
	Streptococcus pseudopneumoniae
	Streptococcus pseudoporcinus
	Streptococcus pyogenes
	Streptococcus ratti
	Streptococcus salivarius
	*Streptococcus salivarius*_ssp_*thermophilus*
	Streptococcus sanguinis
	Streptococcus sinensis
	Streptococcus sobrinus
	Streptococcus sp
	Streptococcus suis
	Streptococcus thoraltensis
	Streptococcus uberis
	Streptococcus urinalis
	Streptococcus vestibularis
