# Supplementary material for: Species identification by MALDI-TOF MS and gap PCR–RFLP of non-aureus Staphylococcus, Mammaliicoccus, and Streptococcus spp. associated with sheep and goat mastitis
Source: Vet Res. 2022 Oct 15;53:84. doi: 10.1186/s13567-022-01102-4 (PMC9569034; doi:10.1186/s13567-022-01102-4)
Supplement: Supplementary file 3 — Additional file 3: Restriction fragment length polymorphism (RFLP) pattern of PCR products of the gap gene obtained after digestion with AluI and used for Staphylococcus species assignment. [file 13567_2022_1102_MOESM3_ESM.docx]

**Additional file 3. Restriction fragment length polymorphism (RFLP) pattern of PCR products of the *gap* gene obtained after digestion with *Alu*I and used for *Staphylococcus* species assignment.** Fragments were separated by 12% NuPAGE gel. Lane 1, *Staphylococcus* (*Staph.*) *epidermidis* ATCC 35983; lane 2, *Staph. xylosus* ATCC 29971^T^; lane 3, *Staph. saprophyticus* ATCC 15305^T^; lane 4, *Staph. capitis* ATCC 27840^T^; lane 5, *Staph. haemolyticus* ATCC 29970^T^; lane 6, *Staph. simulans* ATCC 27848^T^; lane 7, *Staph. warneri* ATCC 27836^T^; lane 8, *Staph. arlettae* ATCC 43957^T^; lane 9, *Staph. chromogenes* ATCC 43764^T^; lane 10, *Staph. equorum* ATCC 43958^T^; lane 11, *Staph. caprae* ATCC 35538^T^; lane 12, *Staph. sciuri* ATCC 29062^T^; lane 13, *Staph. hyicus* ATCC 11249^T^, and lane 14, *Staph. intermedius* ATCC 29663^T^. M, Marker VIII (Roche).

**

**
