# Supplementary material for: Species identification by MALDI-TOF MS and gap PCR–RFLP of non-aureus Staphylococcus, Mammaliicoccus, and Streptococcus spp. associated with sheep and goat mastitis
Source: Vet Res. 2022 Oct 15;53:84. doi: 10.1186/s13567-022-01102-4 (PMC9569034; doi:10.1186/s13567-022-01102-4)
Supplement: Supplementary file 4 — Additional file 4: Restriction fragment length polymorphism (RFLP) pattern of PCR products of the gap gene obtained after digestion with AluI and used for Streptococcus species assignment. [file 13567_2022_1102_MOESM4_ESM.docx]

**Additional file 4. Restriction fragment length polymorphism (RFLP) pattern of PCR products of the *gap* gene obtained after digestion with *Alu*I and used for *Streptococcus* species assignment.** Fragments were separated by 12% NuPAGE gel. Lane 1, *Streptococcus uberis* ATCC 700407; lane 2, *Strep. dysgalactiae* subsp. *dysgalactiae* ATCC 43078^T^; lane 3, Strep. *dysgalactiae* subsp. *equisimilis* DSM 23147^T^; lane 4, *Strep. agalactiae* ATCC 13813^T^; lane 5, *Strep. gallolyticus* subsp. *gallolyticus* ATCC 49475; lane 6, *Strep. equi* subsp. *zooepidemicus* NCTC 6180; and lane 7, *Strep. suis* ATCC 43765. M, Marker VIII (Roche).
