# Supplementary material for: Species identification by MALDI-TOF MS and gap PCR–RFLP of non-aureus Staphylococcus, Mammaliicoccus, and Streptococcus spp. associated with sheep and goat mastitis
Source: Vet Res. 2022 Oct 15;53:84. doi: 10.1186/s13567-022-01102-4 (PMC9569034; doi:10.1186/s13567-022-01102-4)
Supplement: Supplementary file 6 — Additional file 6: Genomic sequence of the gap gene and sequence similarity data for staphylococci. [file 13567_2022_1102_MOESM6_ESM.pdf]

Isolate N. 18 (100979) - *Staphylococcus muscae* (Identity ID 92.79%)

GTAGAAATTGGTCGTTTAGCATTTAGAAGAAATTCAAGATGTAGAAAATATCGAAAGTTGTAGCAGTTAACGATTTAA
CAGACGATGACATGCTTGCACACTTATTAAAATATGACACAATGCAAGGACGTTTTACTGAAGAAGTAGAAGTAATTG
ACGGTGGGTTCCGCGTAAACGGTAAAGAAGTAAAATCATTCTCAGAACCAGAACCTTCAAAATTACCATGGGCTGACT
TAGGTGTAGATGTAGTGCTTGAATGTACAGGTTTCTTCACATCTAAAGAAAAAGCTGAAGCACACATTGAAGCAGGTG
CTAAAAAAGTATTAATCTCTGCTCCTGGTCAAGGCGACCTTAAAACAATCGTATACAATGTTAACCACGAATTATTAGA
CGGTTCTGAAACAGTAGTTTCTGGTGCATCATGTACTACAAACTCACTTGCACCAGTAGCTAAAACTTTACAAGATTCA
TTCGGTATCGTTGAAGGTCTTATGACTACAATTCACGCATACACTGGTGACCAAAATACACAAGACTCACCACACCGTA
AAGGCGACAAACGTCGTGCGCGTGCAGCTGCAGAAAACATCATTCCTAACTCAACAGGTGCTGCTAAAGCAATCGGT
CTTGTAATCCCAGAAATTGCTGGTAAATTAGACGGTGGCGCACAACGTGTACCAGTAGCAACAGGTTCATTAACTGAA
TTAACAGTAGTTCTTGAAAAAGAAGTAACAGTTGAAGAAGTTAACCAAGCAATGAAAGACGCAACTAACGAATCATTC
GGTTACACTGAAGACGAAATCGTATCTTCTGACGTAGTAGGTATGACATTCGGTGCATTATTCGATGCAACACA

Description	Scientific Name	Max Score	Total Score	Query Cover	E value	Per. Ident	Acc. Len	Accession
Staphylococcus muscae glyceraldehyde-3-phosphate dehydrogenase (gap) gene, partial cds	Staphylococcus ...	1323	1323	100%	0.0	92.79%	931	DQ321694.1
Staphylococcus muscae strain ATCC 49910 chromosome, complete genome	Staphylococcus ...	1301	1301	100%	0.0	92.36%	2095131	CP027848.1
Staphylococcus muscae strain NCTC13833 genome assembly, chromosome: 1	Staphylococcus ...	1301	1301	100%	0.0	92.36%	2095116	LT906464.1
Staphylococcus chromogenes glyceraldehyde-3-phosphate dehydrogenase (gap) gene, partial cds	Staphylococcus ...	1123	1123	100%	0.0	88.88%	931	AF495478.1
Staphylococcus chromogenes strain 20B chromosome, complete genome	Staphylococcus ...	1118	1118	100%	0.0	88.77%	2424566	CP031471.1
Staphylococcus chromogenes strain 1401 chromosome, complete genome	Staphylococcus ...	1112	1112	100%	0.0	88.67%	2350748	CP046028.1

Isolate N. 22 (82394) - *Staphylococcus simulans* (Identity ID 99.89%)

AGAATTGGTCGTTTAGCATTCAGAAGAATTCAAGATGTTGAAGGTATCGATGTAGTAGCAGTAAACGACTTAACAGAT
GATGAAATGTTAGCACACTTATTAAAATACGATACAATGCAAGGACGTTTCACTGAAGAAGTTGAAGTTGTAGATGGC
GGATTCCGCGTGAATGGTAAAGAAGTTAAATCATTCGAAGAACCAGATGCAAGCAAATTACCTTGGAAAGATTTAGA
CATTGATGTCGTATTAGAATGTACTGGTTTCTACACTAGCGACGAAAAAGCACAAGCTCACATTGACGCAGGTGCTAA
AAAAGTATTAATCTCTGCACCAGCAACTGGTGACGTTAAAACAATTGTTTATAACGTAAACCAAGATACTTTAGACAGC
TCTGACGTAATCGTTTCAGGTGCTTCTTGTACTACAAACTCACTTGCTCCAGTAGCAAAAGTATTAAATGACAGCTTCG
GTTTAGTAGAAGGTTTCATGACTACTATCCACGCTTACACTGGTGACCAAAATACTCAAGACGGTCCACACAGAAAAG
GCGACAAACGTCGTGCACGTGCAGCAGCTGAAAACATCGTTCCTAACTCAACTGGTGCTGCTAAAGCAATCGGTAAA
GTAATTCCTGAAATCGACGGAAAATTAGACGGTGGCGCTCAACGTGTTCCTGTAGCAACTGGTTCATTAACTGAATTA
ACAGTTGTATTAGACAAAGACGTAACAATCGAAGAAGTAAACGAAGCTATGAAAGCAGCTTCTAACGAATCATTCGG
TTACAACGAAGACGAAATCGTATCTTCAGACGTAGTTGGTATGACATTCGGTTCATTATTCGATGCAACTCAAACTCGT
GTTATGACTGTATCC

Description	Scientific Name	Max Score	Total Score	Query Cover	E value	Per. Ident	Acc. Len	Accession
Staphylococcus simulans strain MR1 chromosome, complete genome	Staphylococcus ...	1663	1663	100%	0.0	99.89%	2661512	CP015642.1
Staphylococcus simulans strain MR2 chromosome, complete genome	Staphylococcus ...	1663	1663	100%	0.0	99.89%	2680372	CP016157.1
Staphylococcus simulans strain MR4 chromosome, complete genome	Staphylococcus ...	1663	1663	100%	0.0	99.89%	2685102	CP017430.1
Staphylococcus simulans strain MR3 chromosome, complete genome	Staphylococcus ...	1663	1663	100%	0.0	99.89%	2679685	CP017428.1
Staphylococcus simulans glyceraldehyde-3-phosphate dehydrogenase (gap) gene, partial cds	Staphylococcus ...	1663	1663	100%	0.0	99.89%	931	DQ321698.1
Staphylococcus simulans strain NCTC7944 genome assembly, chromosome: 1	Staphylococcus ...	1657	1657	100%	0.0	99.78%	2675237	LR134264.1

Isolate N. 24 (18886) - *Staphylococcus jettensis* (Identity ID 99.29%, Total score 1520)

GTATGATAACGCAAATGTACAGACTTAACTGATGACGAAATGTTAGCTCATTTATTAAAATATGACACTATGCAAGGTC
GCTTCACTAGTGAAGTTGAAGTTATCGATGGTGGTTTCCGTGTAAATGGTAAAGAAGTTAAATCTTACGAAGAACCAG
ATGCAAGCAAATTACCTTGGGGCGATTTAGATATCGACGTAGTATTAGAATGTACTGGTTTCTATACTGATAAAGAAA
AAGCAGAAGCTCACATCAATGCAGGAGCTAAAAAAGTATTAATTTCTGCACCAGCTAAAGGTGACGTTAAAACAATCG
TATTCAACACTAACCACAAAGACTTAGATGGTTCTGAAACAGTAGTATCAGGTGCTTCATGTACTACTAACTCATTAGC
ACCAGTTGCTAAAGTATTAAGCGATGAATTCGGTATCGTTGAAGGTTTAATGACAACTATCCACGCTTACACTGGTGA
CCAAATGACTCAAGACGGTCCTCACAAAAAAGGTGACAAACGTCGTGCTCGTGCAGCAGCTCAAAACATCGTACCTAA
CTCAACAGGTGCAGCTAAAGCTATCGGTAAAGTAATTCCTGAAATCGATGGTAAATTAGACGGTGGTGCACAACGTGT
ACCTGTAGCAACTGGTTCATTAACTGAAGTAACAGTTGTATTAGAAAAAGACGTTACAGTTGAAGACGTTAACAATGC
TATGAAAAACGCTTCAAACGAATCATTCGGTTACACTGAAGACGAAATCGTTTCTTCAGACGTAGTTGGTATGACTTAC
GGTTCATTATTCGATGCTACACAAAACTCGTGTAATGTCAGTTGGTGACCGTCAAATTTAGTTTAAAGTTTGCAGCTTG
GTATTGATAACGAAAAA

Description	Scientific Name	Max Score	Total Score	Query Cover	E value	Per. Ident	Acc. Len	Accession
Staphylococcus jettensis strain SEQ258 glyceraldehyde-3-phosphate dehydrogenase (gap) gene, partial cds	Staphylococcus ...	1520	1520	96%	0.0	99.29%	903	JN092108.1
Staphylococcus jettensis strain SEQ257 glyceraldehyde-3-phosphate dehydrogenase (gap) gene, partial cds	Staphylococcus ...	1506	1506	96%	0.0	98.93%	905	JN092107.1
Staphylococcus jettensis strain SEQ256 glyceraldehyde-3-phosphate dehydrogenase (gap) gene, partial cds	Staphylococcus ...	1506	1506	96%	0.0	98.93%	907	JN092106.1
Staphylococcus jettensis strain SEQ255 glyceraldehyde-3-phosphate dehydrogenase (gap) gene, partial cds	Staphylococcus ...	1506	1506	96%	0.0	98.93%	906	JN092105.1
Staphylococcus jettensis strain SEQ110 glyceraldehyde-3-phosphate dehydrogenase (gap) gene, partial cds	Staphylococcus ...	1504	1504	96%	0.0	98.93%	903	JN092110.1
Staphylococcus jettensis strain SEQ259 glyceraldehyde-3-phosphate dehydrogenase (gap) gene, partial cds	Staphylococcus ...	1504	1504	96%	0.0	98.93%	900	JN092109.1
Staphylococcus petrasii subsp. petrasii strain CCM 8418 glyceraldehyde-3-phosphate dehydrogenase (gap) g...	Staphylococcus ...	1502	1502	94%	0.0	99.40%	880	JX139895.1
Staphylococcus jettensis strain SEQ036 glyceraldehyde-3-phosphate dehydrogenase (gap) gene, partial cds	Staphylococcus ...	1498	1498	95%	0.0	98.93%	910	JN092104.1

Isolate N. 26 (62676) - *Staphylococcus hyicus* (Identity ID 98.69%)

GGTAGAATTGGTCGTTTAGCATTTAGAAGAATTCAAGACGTAGAAAAACATTGAGGTAGTAGGCTGTCAAATGATTTA
AACTGACGACGACATGCTTGCACACTTATTAAAAGTATGATACTATGCAAGGACGTTTTACTGAAGAAGTAGATGTAA
TTGATGGTGGTTTCCGCGTAAATGGTAAAGAAGTGAAATCATTCTCTGAACCAGAACCATCTAAATTACCTTGGAAAG
ATTTAGAAGTAGATGTTGTATTAGAATGTACTGGTTTCTTCACATCTAAAGAAAAAGCTGAAGCACACATCGAAGCTG
GTGCTAAAAAAGTTTTAATTTCAGCTCCAGGTACTGGCGATCTTAAAACAATCGTATACAACGTTAACCATGAAGAATT
AGACGGTTCAGAAACAGTTGTTTCAGGTGCATCTTGTACTACAAACTCATTAGCACCAGTAGCGAAAACATTACACGA
TGAATTTGGCATCGTTGAAGGTTTAATGACTACGATTCACGCTTACACAGGTGACCAAAATACACAAGATTCACCTCAC
AGAAAAGGTGACAAACGTCGTGCACGTGCAGCAGCTGAAAACATCATCCCTAACTCAACTGGTGCTGCAAAAGCAAT
CGGTTTAGTTATTCCAGAAATCGCTGGTAAATTAGATGGTGGGGCACAACGTGTACCAGTTGCTACAGGTTCATTAAC
AGAATTAACTGTAGTTTTAGAAAAAGAAGTTTCTGTTGAAGAAGTAAACAATGCTATGAAAAATGCAACGAATTATTG
TTTTGGTAGA

Description	Scientific Name	Max Score	Total Score	Query Cover	E value	Per. Ident	Acc. Len	Accession
Staphylococcus hyicus strain UDI 129 glyceraldehyde-3-phosphate dehydrogenase (gap) gene, partial cds	Staphylococcus ...	1626	1626	100%	0.0	98.69%	926	FJ578006.1
Staphylococcus agnetis strain 1379 chromosome, complete genome	Staphylococcus ...	1622	1622	99%	0.0	98.69%	2449249	CP045927.1
Staphylococcus agnetis strain 12B chromosome, complete genome	Staphylococcus ...	1622	1622	99%	0.0	98.69%	2345021	CP031266.1
Staphylococcus agnetis strain 908 chromosome, complete genome	Staphylococcus ...	1622	1622	99%	0.0	98.69%	2474434	CP009623.1
Staphylococcus agnetis strain G4 chromosome	Staphylococcus ...	1622	1622	99%	0.0	98.69%	2489066	CP081015.1
Staphylococcus hyicus strain HY glyceraldehyde-3-phosphate dehydrogenase (gap) gene, partial cds	Staphylococcus ...	1520	1520	100%	0.0	96.63%	931	JQ728499.1
Staphylococcus hyicus strain HDSY glyceraldehyde-3-phosphate dehydrogenase (gap) gene, partial cds	Staphylococcus ...	1482	1482	100%	0.0	95.87%	931	JQ728497.1
[illegible]	[illegible]	[illegible]	[illegible]	[illegible]	[illegible]	[illegible]	[illegible]	[illegible]

Isolate N. 27 (63653) - *Staphylococcus pseudintermedius* (Identity ID 98.81%)

GGTTTTTGGTGAGAAATTGGTCGTTTAGGCATTTAGAAGAATTCAAGATGTGGAAAAATATCGACGTTGTAGCAGTAA
AATGATTTAACAGACGACGATATGCTTGCACACTTATAAAAATATGACTCAACACAAGGTCGTTTTACTGAAGAAGTA
GAAGTAATTGACGGTGGTTTTCCGTGTAAATGGTAAAGAAGTGAAATCATTCTCTGAACCAGAAACCTAAAAACTTAC
CATGGGGCGAGTTAGACATCGACGTGGTATTAGAATGTACGGGTTTCTTCACTGATAAAGAAAAAGCTGAAGCACAC
ATCGAAGCAGGCGCGAAAAAAGTATTAATTTCTGCACCAGCTAAAGGTGACCTTAAAACAGTTGTATATAACGTTAAC
CACGAAATTTTAGATGGTACTGAAACAGTTGTTTCTGGTGCTTCATGTACAACAAACTCATTAGCACCTGTTGCCAAAA
CTTTACAAGACAACTTTGGTATCGTTGAAGGTTTAATGACAACAATTCACGCTTACACTGGTGACCAAAACACATTAGA
CGCACCTCACAGAAAAGGTGACAAACGTCGTGCGCGTGCAGCTGCTGAAAACATTATCCCTAACTCAACTGGTGCTGC
GAAAGCAATCGGTTTAGTTATTCCTGAAATCGATGGTAAATTAGACGGTGGTGCACAACGTGTTCCTGTAGCAACTGG
TTCATTAACTGAATTAACAGTTGTATTAGAAAAAGAAGTTTCAGTTGAAGAAGTTAACAAAGT

	Description	Scientific Name	Max Score	Total Score	Query Cover	E value	Per. Ident	Acc. Len	Accession
☑	Staphylococcus pseudintermedius strain MAD401 chromosome	Staphylococcus pseudintermedius	1639	1639	99%	0.0	98.81%	2883513	CP039742.1
☑	Staphylococcus pseudintermedius strain 063228 chromosome, complete genome	Staphylococcus pseudintermedius	1639	1639	99%	0.0	98.81%	2766566	CP015626.1
☑	Staphylococcus pseudintermedius strain ME4692 chromosome	Staphylococcus pseudintermedius	1633	1633	98%	0.0	98.91%	2750501	CP039747.1
☑	Staphylococcus pseudintermedius strain AH18 chromosome, complete genome	Staphylococcus pseudintermedius	1633	1633	98%	0.0	98.91%	2623199	CP030374.1
☑	Staphylococcus pseudintermedius strain 5912, complete genome	Staphylococcus pseudintermedius	1633	1633	99%	0.0	98.70%	2531995	CP009120.1
☑	Staphylococcus pseudintermedius strain 53_88 chromosome	Staphylococcus pseudintermedius	1633	1633	99%	0.0	98.70%	2593941	CP035740.1
☑	Staphylococcus pseudintermedius strain 53_60 chromosome	Staphylococcus pseudintermedius	1633	1633	98%	0.0	98.91%	2615959	CP035741.1
☑	Staphylococcus pseudintermedius strain 51_92 chromosome	Staphylococcus pseudintermedius	1633	1633	99%	0.0	98.70%	2512363	CP035742.1
☑	Staphylococcus pseudintermedius strain 49_44 chromosome	Staphylococcus pseudintermedius	1633	1633	98%	0.0	98.91%	2584763	CP035743.1

Isolate N. 32 (10255) - *Staphylococcus devriesei* (Identity ID 99.77%)

TTTTTGTAGAATTGGTCGTTTAGCATTTAGAAGAATTCAAGACGTAGAAGGTATTGAAGTAGTTGCAGTAAACGACTT
AACTGACGACGAAATGTTAGCTCATTTATTAAAATATGACACTATGCAAGGACGCTTCACTGGTGAAGTTGAAGTCGT
TGATGGTGGTTTCCGCGTAAATGGTAAAGAAGTTAAATCATACGAAGAACCAGACGCAAGCAAATTACCTTGGGGCG
ATTTAGATATCGACGTAGTATTAGAATGTACTGGTTTCTATACAGATAAAGAAAAAGCAGAAGCACACATTAATGCAG
GAGCTAAAAAAGTATTAATCTCTGCTCCAGCTAAAGGTGACGTTAAAACAATCGTATATAACACTAACCACAGCGACT
TAGACGGTTCAGAAACAGTTGTTTCAGGTGCTTCATGTACTACTAACTCATTAGCACCAGTTGCAAAAGTATTAAGTGA
TGAATTCGGTATCGTTGAAGGTTTAATGACTACTATCCATGCATACACTGGTGACCAAATGACTCAAGACGGTCCTCAT
AAAAAAGGCGACAAACGTCGTGCACGTGCAGCAGCTCAAAACATCGTACCTAACTCAACAGGTGCTGCTAAAGCTAT
CGGTAAAGTTATTCCTGAAATCGATGGTAAATTAGACGGCGGTGCACAACGTGTACCAGTAGCAACTGGTTCATTAAC
TGAAGTAACAGTTGTATTAGAAAAAGACGTTTCAGTTGAAGACGTTAACAATGCAATGAAAAATGCTTCAAACGAATC
ATTCGGTTACACTGAAGACGAAATCGTATCTTCAGACGTAGTAGGTATGACTTACGGTTCATTATTCGACGCTACACAA
ACTCGTGTAATGTCAGTAGGCGACCGTC

	Description	Scientific Name	Max Score	Total Score	Query Cover	E value	Per. Ident	Acc. Len	Accession
☑	Staphylococcus devriesei strain CCM 7896 glyceraldehyde-3-phosphate dehydrogenase (gap) gene, partial cds	Staphylococcus ...	1615	1615	96%	0.0	99.77%	880	JX174278.1
☑	Staphylococcus devriesei strain F101 glyceraldehyde-3-phosphate dehydrogenase (gap) gene, partial cds	Staphylococcus ...	1511	1511	90%	0.0	99.76%	824	KM251711.1
☑	Staphylococcus jettensis strain SEQ256 glyceraldehyde-3-phosphate dehydrogenase (gap) gene, partial cds	Staphylococcus ...	1419	1419	99%	0.0	95.02%	907	JN092106.1
☑	Staphylococcus taiwanensis strain NTUH-S172 chromosome, complete genome	Staphylococcus ...	1417	1417	100%	0.0	94.73%	2517683	CP058667.1
☑	Staphylococcus jettensis strain SEQ110 glyceraldehyde-3-phosphate dehydrogenase (gap) gene, partial cds	Staphylococcus ...	1417	1417	99%	0.0	95.01%	903	JN092110.1
☑	Staphylococcus jettensis strain SEQ257 glyceraldehyde-3-phosphate dehydrogenase (gap) gene, partial cds	Staphylococcus ...	1417	1417	99%	0.0	95.01%	905	JN092107.1

Isolate N. 35 (22815) - *Staphylococcus pseudoxylosus* (Identity ID 99.78%)

GGTAGAATTGGTCGTTTAGCATTCAGAAGAATTCAAAACGTTGATGGAATTGACGTAGTAGCAGTAAATGATTTAACA
GATGACGAAATGTTAGCACATTTATTAAAATATGACACTATGCAAGGACGCTTCACAGGAGAAGTTGAAGTTGAAATG
ACGGTTTCCGTGTTAACGGACAAGAAGTAAAATCATTCTCTGAGCCAGAACCAAGTAAATTACCTTGGAAAGACTTAG
ACATTGATGTTGTATTAGAATGTACTGGTTTCTTCGCTGATAAAGAAAAAGCAGAAGCTCATATTGAAGCAGGTGCTA
AAAAAGTATTAATCTCTGCTCCAGCTACTGGTGACTTAAAAACAATCGTTTATAACACAAACCACCAAGAATTAGATGG
TTCAGAAACAGTAGTTTCAGGTGCTTCATGTACTACTAACTCATTAGCTCCAGTTGCAAAAGTATTAAACGATGACTTC
GGTTTAGTAGAAGGTTTCATGACTACAATCCATGCTTACACTGGTGACCAAAGCACACAAGATGCTCCACACAGAAAA
GGCGACAAACGTCGTGCGCGTGCAGCAGCTGAAAACATTATTCCTAACTCAACTGGTGCTGCTAAAGCAATTGGCCTA
GTAATCCCTGAAATCGATGGTAAATTAGACGGTGGAGCGCAACGTGTTCCTGTAGCTACTGGTTCTTTAACTGAATTA
ACAGTTGTATTAGAGAAAAATGTAAGTATTGAAGACGTTAATGCTTCAATGAAAAATGCATCAAACGAATCATTCGGT
TACACTGAAGATGAAATCGTTTCTTCTGACGTAATTGGTATGACTTACGGTTCATTATTCGATGCAACACAAACACGTG
TAA

Description	Scientific Name	Max Score	Total Score	Query Cover	E value	Per. Ident	Acc. Len	Accession
Staphylococcus pseudoxylosus strain 14AME19 chromosome, complete genome	Staphylococcus ...	1674	1674	99%	0.0	99.78%	2910290	CP068712.1
Staphylococcus xylosus strain LZ glyceraldehyde-3-phosphate dehydrogenase (gap) gene, partial cds	Staphylococcus ...	1570	1570	93%	0.0	99.65%	860	JQ728502.1
Staphylococcus xylosus strain SH-2 glyceraldehyde-3-phosphate dehydrogenase (gap) gene, partial cds	Staphylococcus ...	1563	1563	93%	0.0	99.53%	859	JQ728507.1
Staphylococcus xylosus strain JM glyceraldehyde-3-phosphate dehydrogenase (gap) gene, partial cds	Staphylococcus ...	1522	1522	93%	0.0	98.94%	862	JQ728501.1
Staphylococcus xylosus partial gap gene for glyceraldehyde-3-phosphate dehydrogenase, strain C8	Staphylococcus ...	1493	1493	89%	0.0	99.75%	815	LK023497.1

Isolate N. 36 (6441) - *Staphylococcus haemolyticus* (Identity ID 98.91%)

TTTTTGGTAGAAATTGGTCGTTTAGCATTTAGAAGAAATTCAAGACGTAGAAGGTATTGAAGTAGTTGCAGTAAACGA
CTTAACAGACGACGAAATGTTAGCTCATTTATTAAAATATGACACTATGCAAGGTCGTTTTACAGGAGAAGTTGAAGT
TATTGATGGTGGTTTCCGCGTAAATGGTAAAGAAGTTAAATCATACGAAGAACCAGATGCAAGCAAATTACCTTGGG
GCGATTTAGATATCGACGTAGTATTAGAATGTACTGGTTTCTATACAGATAAAGAAAAAGCTGAAGCACACATCAATG
CAGGTGCTAAAAAAGTATTAATCTCTGCACCAGCTAAAGGTGATGTTAAAACAATCGTATTCAACACTAACCACAATG
ACTTAGATGGTTCAGAAACAGTTGTTTCAGGTGCATCATGTACTACTAACTCATTAGCACCAGTTGCTAAAGTGTTAAG
TGACGAATTTGGTTTAGTTGAAGGTTTAATGACAACTATTCACGCATACACTGGTGACCAAATGACTCAAGACGGTCC
ACATAAAAAAGGCGATAAACGTCGTGCGCGTGCAGCAGCTCAAAACATCGTACCAAACTCAACAGGTGCTGCAAAAG
CTATCGGTAAAGTTATTCCTGAAATCGATGGTAAATTAGACGGTGGTGCTCAACGTGTACCAGTTGCTACAGGTTCATT
AACTGAAGTAACAGTTGTATTAGAAAAAGACGTTACTGTTGAAGACGTTAACAAAGCAATGAAAAACGCTTCAAACG
AATCATTCGGTTACACTGAAGACGAAATCGTTTCTTCAGACGTAGTTGGTATGACTTACGGTTCATTATTCGATGCTAC
TCAAACTCGTGTAATG

Description	Scientific Name	Max Score	Total Score	Query Cover	E value	Per. Ident	Acc. Len	Accession
Staphylococcus haemolyticus strain GDY8P80P chromosome, complete genome	Staphylococcus ...	1639	1639	99%	0.0	98.91%	2715577	CP063443.1
Staphylococcus haemolyticus strain SE2.14 chromosome, complete genome	Staphylococcus ...	1600	1600	99%	0.0	98.15%	2323230	CP084235.1
Staphylococcus haemolyticus strain SE3.8 chromosome, complete genome	Staphylococcus ...	1600	1600	99%	0.0	98.15%	2323407	CP084229.1
Staphylococcus haemolyticus strain NY5 chromosome, complete genome	Staphylococcus ...	1600	1600	99%	0.0	98.15%	2472146	CP078159.1
Staphylococcus haemolyticus strain SE3.9 chromosome, complete genome	Staphylococcus ...	1600	1600	99%	0.0	98.15%	2323296	CP049091.1
Staphylococcus haemolyticus strain SCAID URN1-2019 chromosome	Staphylococcus ...	1589	1589	99%	0.0	97.93%	2256190	CP052055.1
Staphylococcus haemolyticus isolate Staphylococcus haemolyticus K8 genome assembly, chromosome: I	Staphylococcus ...	1589	1589	99%	0.0	97.93%	2426012	LT963441.1
Staphylococcus haemolyticus strain 12b chromosome, complete genome	Staphylococcus ...	1589	1589	99%	0.0	97.93%	2339810	CP071505.1
Staphylococcus haemolyticus strain 7b chromosome, complete genome	Staphylococcus ...	1589	1589	99%	0.0	97.93%	2341142	CP071508.1
Staphylococcus haemolyticus strain 1b chromosome, complete genome	Staphylococcus ...	1589	1589	99%	0.0	97.93%	2339731	CP071512.1
Staphylococcus haemolyticus JCSC1435 DNA, complete genome	Staphylococcus ...	1589	1589	99%	0.0	97.93%	2685015	AP006716.1
Staphylococcus haemolyticus strain VB5326 chromosome, complete genome	Staphylococcus ...	1583	1583	99%	0.0	97.83%	2699292	CP045137.2
Staphylococcus haemolyticus strain VB19458 chromosome, complete genome	Staphylococcus ...	1583	1583	99%	0.0	97.83%	2699210	CP045187.2

Isolate N. 39 (22) - *Staphylococcus chromogenes* (Identity ID 98.75%)

CGTGTTTGAGTTGCATCAAATAATGCACCAAAAGTCATGCCTACAACATCTGAAGATACGATTTCGTCTTCAGTGTAAC
CGAATGATTCGTTAGTTGCATTTTTCATTGCATTGTTAACGTCTTCTACTGATACTTCTTTATCTAAAACAACTGTTAATT
CAGTTAATGAACCTGTTGCTACTGGTACACGTTGTGCGCCACCGTCTAATTTTCCATCAATTTCAGGGATAACTAAACC
GATTGCTTTCGCAGCACCTGTTGAGTTAGGAATAATGTTTTCTGCAGCTGCACGTGCACGACGTTTGTCACCTTTTCTG
TGTGGTGAGTCTTGTGTATTTTGGTCACCAGTGTATGCGTGAATTGTAGTCATTAAACCTTCAACGATACCAAATTCAT
CGTTTAAAGTTTTTGCTACTGGTGCTAATGAGTTTGTTGTACAAGAAGCACCTGATACAACTGTTTCAGAACCGTCTAA
TTCTTCATGGTTGACATTATATACGATTGTTTTAAGATCGCCAGTTCCTGGTGCAGAAATTAATACTTTTTTAGCACCTG
CTTCAATGTGAGTTTCTGCTTTTTCTTTTGATGTAAAGAAACCTGTACATTCTAAAACAACATCTACGTCAAGATCTTTCC
ATGGTAATTTTGATGGTTCTGGTTCAGAGAATGATTTCACTTCTTTACCATTTACGCGGAAACCACCATCAATTACATCT
ACTTCTTCAGTAAAACGACCTTGCATTGTGTCATATTTCAATAAATGTGCAAGCATATCGTCGTCTGTTAAATCGTTTAC
AGCTACAACCTCAATATTTTCTACGTCTTGAATTCTTCTGAATGCTAAACGAC

	Description	Scientific Name	Max Score	Total Score	Query Cover	E value	Per. Ident	Acc. Len	Accession
☑	Staphylococcus chromogenes strain 20B chromosome, complete genome	Staphylococcus ...	1561	1561	100%	0.0	98.75%	2424566	CP031471.1
☑	Staphylococcus chromogenes glyceraldehyde-3-phosphate dehydrogenase (gap) gene, partial cds	Staphylococcus ...	1561	1561	100%	0.0	98.75%	931	AF495478.1
☑	Staphylococcus chromogenes strain 1401 chromosome, complete genome	Staphylococcus ...	1555	1555	100%	0.0	98.64%	2350748	CP046028.1
☑	Staphylococcus chromogenes strain 17A chromosome, complete genome	Staphylococcus ...	1555	1555	100%	0.0	98.64%	2351540	CP031274.1
☑	Staphylococcus chromogenes strain 34B chromosome, complete genome	Staphylococcus ...	1550	1550	100%	0.0	98.52%	2369172	CP031470.1
☑	Staphylococcus chromogenes strain SG glyceraldehyde-3-phosphate dehydrogenase (gap) gene, partial cds	Staphylococcus ...	1548	1548	99%	0.0	98.63%	892	JQ728506.1

Isolate N. 49 (17963) - *Staphylococcus haemolyticus* (Identity ID 99.07%)

GGTAGAATTGGTCGTTTAGCATTTAGAAGAAATTCAAAGACGTAGAAAGGTATTGAAGTAGTTGCAGTAAACGACTT
AACAGACGACGAAATGTTAGCTCATTTATTAAAATATGACACTATGCAAGGTCGTTTTACAGGAGAAGTTGAAGTTAT
TGATGGTGGTTTCCGCGTAAATGGTAAAGAAGTTAAATCATACGAAGAACCAGATGCAAGCAAATTACCTTGGGGCG
ATTTAGATATCGACGTAGTATTAGAATGTACTGGTTTCTATACAGATAAAGAAAAAGCTGAAGCACACATCAATGCAG
GTGCTAAAAAAGTATTAATCTCTGCACCAGCTAAAGGTGATGTTAAAACAATCGTATTCAACACTAACCACAATGACTT
AGATGGTTCAGAAACAGTTGTTTCAGGTGCATCATGTACTACTAACTCATTAGCACCAGTTGCTAAAGTGTTAAGTGAC
GAATTTGGTTTAGTTGAAGGTTTAATGACAACTATTCACGCATACACTGGTGACCAAATGACTCAAGACGGTCCACAT
AAAAAAGGCGATAAACGTCGTGCGCGTGCAGCAGCTCAAAACATCGTACCAAACTCAACAGGTGCTGCAAAAGCTAT
CGGTAAAGTTATTCCTGAAATCGATGGTAAATTAGACGGTGGTGCTCAACGTGTACCAGTTGCTACAGGTTCATTAAC
TGAAGTAACAGTTGTATTAGAAAAAGACGTTACTGTTGAAGACGTTAACAAAGCAATGAAAAACGCTTCAAACGAATC
ATTCGGTTACACTGAAGACGAAATCGTTTCTTCAGACGTAGTTGGTATGACTTACGGTTCATTATTCGATGCTACTCAA
A

	Description	Scientific Name	Max Score	Total Score	Query Cover	E value	Per. Ident	Acc. Len	Accession
☑	Staphylococcus haemolyticus strain GDY8P80P chromosome, complete genome	Staphylococcus ...	1574	1574	100%	0.0	98.98%	2715577	CP063443.1
☑	Staphylococcus haemolyticus partial gap gene for glyceraldehyde-3-phosphate dehydrogenase, strain C4	Staphylococcus ...	1539	1539	97%	0.0	99.07%	864	LK023494.1
☑	Staphylococcus haemolyticus strain SE2.14 chromosome, complete genome	Staphylococcus ...	1535	1535	100%	0.0	98.18%	2323230	CP084235.1
☑	Staphylococcus haemolyticus strain SE3.8 chromosome, complete genome	Staphylococcus ...	1535	1535	100%	0.0	98.18%	2323407	CP084229.1
☑	Staphylococcus haemolyticus strain NY5 chromosome, complete genome	Staphylococcus ...	1535	1535	100%	0.0	98.18%	2472146	CP078159.1
☑	Staphylococcus haemolyticus strain SE3.9 chromosome, complete genome	Staphylococcus ...	1535	1535	100%	0.0	98.18%	2323296	CP049091.1

Isolate N. 232 (10511) - *Staphylococcus epidermidis* (Identity ID 98.48%)

GGTAGAATTTGGTCGTTTAGCATTTCAGAAAGAATTCAAGATGTAGAAGGTCTTGAAAGTAGTTGCAGTTAAACGACT
TAACAGATGACGATATGTTAGCTCATTTATTAAAATACGATACTATGCAAGGTCGTTTCACTGGAGAAGTTGAAGTTAT
CGAAGGTGGATTCCGTGTTAACGGTAAAGAAATTAAATCATTCGATGAACCAGATGCTGGTAAATTACCATGGGGCG
ATTTAGATATCGACGTAGTATTAGAATGTACTGGTTTCTATACTGATAAAGAAAAAGCACAAGCTCACATCGATGCAG
GTGCTAAAAAAGTATTAATCTCAGCTCCAGCTAAAGGTGATGTAAAAACAATCGTATTCAACACTAACCATGACACATT
AGATGGTTCAGAAACAGTTGTTTCAGGTGCTTCTTGTACTACTAACTCATTAGCACCAGTTGCAAAAGTTTTAAGTGAT
GAATTCGGTTTAGTTGAAGGTTTCATGACTACAATTCACGCTTACACTGGTGACCAAAATACACAAGACGCACCTCACA
GAAAAGGTGACAAACGTCGTGCACGTGCAGCAGCAGAAAATATTATCCCTAACTCAACAGGTGCTGCTAAAGCTATC
GGTAAAGTTATTCCAGAAATCGATGGTAAATTAGACGGTGGAGCACAACGTGTTCCAGTTGCTACTGGTTCTTTAACT
GAATTAACTGTAGTATTAGACAAACAAGATGTAACTGTTGACCAAGTTAACAGTGCTATGAAACAAGCTTCAGACGAA
TCATTCGGTTACACTGAAGACGAAATCGTATCTTCTGATATCGTTGGTATGACTTACGGTTCATTATTCGATGCGACTC
AAACTCGTGTTAGACTA

Description	Scientific Name	Common Name	Taxid	Max Score	Total Score	Query Cover	E value	Per. Ident	Acc. Len	Accession
Staphylococcus epidermidis strain SESURV_p2_0614 chromosome	Staphyl...	NA	1282	1646	1646	99%	0.0	99.23%	2523089	CP043788.1
Staphylococcus epidermidis strain TMDU-128 chromosome, complete genome	Staphyl...	NA	1282	1646	1646	99%	0.0	99.23%	2551919	CP093170.1
Staphylococcus epidermidis strain CBPA-ST-11003 chromosome, complete genome	Staphyl...	NA	1282	1646	1646	99%	0.0	99.23%	2566999	CP071992.1
Staphylococcus epidermidis strain HD104-2 chromosome, complete genome	Staphyl...	NA	1282	1646	1646	99%	0.0	99.23%	2453988	CP053007.1
Staphylococcus epidermidis strain 14.1.R1 chromosome, complete genome	Staphyl...	NA	1282	1618	1618	99%	0.0	98.69%	2572575	CP018842.1
Staphylococcus epidermidis strain BYQ glyceraldehyde-3-phosphate dehydrogenase (gap) gene, par...	Staphyl...	NA	1282	1616	1616	100%	0.0	98.48%	927	JQ728491.1
Staphylococcus epidermidis strain sep1 chromosome	Staphyl...	NA	1282	1613	1613	99%	0.0	98.58%	2536282	CP101316.1

Isolate N. 264 (85364) - *Staphylococcus haemolyticus* (Identity ID 98.26%)

TTTTGGTAGAATTGGTCGTTTAGCATTTAGAAGAATTCAAGACGTAGAAGGTATTGAAGTAGTGCAGTAAACGACTTA
ACAGACGACGAAATGTTAGCTCATTTATTAAAATATGACACTATGCAAGGTCGTTTCACAGGAGAAGTTGAAGTTATT
GATGGTGGTTTCCGCGTAAATGGTAAAGAAGTTAAATCATACGAAGAACCAGATGCAAGCAAATTACCTTGGGGCGA
TTTAGATATCGACGTAGTATTAGAATGTACTGGTTTCTATACAGATAAAGAAAAAGCAGAAGCACACATCAATGCAGG
TGCTAAAAAAGTATTAATCTCTGCACCAGCTAAAGGTGATGTTAAAACAATCGTATTCAACACTAACCACAATGACTTA
GATGGTTCAGAAACAGTTGTTTCAGGTGCATCATGTACTACTAACTCATTAGCACCAGTTGCTAAAGTGTTAAGTGAC
GAATTTGGTTTAGTTGAAGGTTTAATGACAACTATTCACGCATACACTGGTGACCAAATGACTCAAGACGGTCCACAT
AAAAAAGGCGACAAACGTCGTGCGCGTGCAGCAGCTCAAAACATCGTACCAAACTCAACAGGTGCTGCAAAAGCTAT
CGGTAAAGTTATTCCTGAAATCGATGGTAAATTAGACGGTGGTGCTCAACGTGTACCAGTTGCTACAGGTTCATTAAC
TGAAGTAACAGTTGTATTAGAAAAAGACGTTACTGTTGAAGACGTTAACAAAGCAATGAAAAACGCTTCAAACGAATC
ATTCGGTTACACTGAAGACGAAATCGTTTGCTTCAGACGTAGTATGAGTATGACTTACGGTTCATTATTCGAGGCTACT

Description	Scientific Name	Max Score	Total Score	Query Cover	E value	Per. Ident	Acc. Len	Accession
Staphylococcus haemolyticus strain GDY8P80P chromosome, complete genome	Staphylococcus ...	1659	1659	99%	0.0	99.35%	2715577	CP063443.1
Staphylococcus haemolyticus strain SE2.14 chromosome, complete genome	Staphylococcus ...	1620	1620	99%	0.0	98.58%	2323230	CP084235.1
Staphylococcus haemolyticus strain SE3.8 chromosome, complete genome	Staphylococcus ...	1620	1620	99%	0.0	98.58%	2323407	CP084229.1
Staphylococcus haemolyticus strain NY5 chromosome, complete genome	Staphylococcus ...	1620	1620	99%	0.0	98.58%	2472146	CP078159.1
Staphylococcus haemolyticus strain SE3.9 chromosome, complete genome	Staphylococcus ...	1620	1620	99%	0.0	98.58%	2323296	CP049091.1
Staphylococcus haemolyticus strain SCAID URN1-2019 chromosome	Staphylococcus ...	1609	1609	99%	0.0	98.37%	2256190	CP052055.1
Staphylococcus haemolyticus isolate Staphylococcus haemolyticus K8 genome assembly, chromosome: I	Staphylococcus ...	1609	1609	99%	0.0	98.37%	2426012	LT963441.1
Staphylococcus haemolyticus strain 12b chromosome, complete genome	Staphylococcus ...	1609	1609	99%	0.0	98.37%	2339810	CP071505.1
Staphylococcus haemolyticus strain 7b chromosome, complete genome	Staphylococcus ...	1609	1609	99%	0.0	98.37%	2341142	CP071508.1
Staphylococcus haemolyticus strain 1b chromosome, complete genome	Staphylococcus ...	1609	1609	99%	0.0	98.37%	2339731	CP071512.1
Staphylococcus haemolyticus JCSC1435 DNA, complete genome	Staphylococcus ...	1609	1609	99%	0.0	98.37%	2685015	AP006716.1
Staphylococcus haemolyticus glyceraldehyde-3-phosphate dehydrogenase (gap) gene, partial cds	Staphylococcus ...	1607	1607	99%	0.0	98.26%	931	DQ321687.1
Staphylococcus haemolyticus strain VB5326 chromosome, complete genome	Staphylococcus ...	1604	1604	99%	0.0	98.26%	2699292	CP045137.2
