# Supplementary material for: Species identification by MALDI-TOF MS and gap PCR–RFLP of non-aureus Staphylococcus, Mammaliicoccus, and Streptococcus spp. associated with sheep and goat mastitis
Source: Vet Res. 2022 Oct 15;53:84. doi: 10.1186/s13567-022-01102-4 (PMC9569034; doi:10.1186/s13567-022-01102-4)
Supplement: Supplementary file 7 — Additional file 7: Genomic sequence of the gap gene and sequence similarity data for streptococci. [file 13567_2022_1102_MOESM7_ESM.pdf]

Isolate N. 3 (15004) - *Streptococcus parauberis* (Identity ID 99.20%)

TAACTAATTGATTTCCATCAACAGTTTTGTACTTTAGTTTGAGTAGCATCGAATAATGAACCGAAAGACATACCAACGA
TATCAGATGATACGATTGGATCTTCAGTGTAACCATATGAATCATTAGCTGCAGCTTTCATTACTGAGTTAATTTCTTCT
ACTGAAGTTTCTTTATTAAGAACTGCTACTAATTCTGTTACTGAACCTGTTGGAACTGGTACACGTTGTGCAGCACCGT
CAAGTTTACCATTTAATTCAGGGATAACAAGACCGATTGCTTTAGCAGCACCAGTTGAGTTAGGAACGATATTGTTAG
CACCAGCACGGGCACGACGTAAGTCACCACCACGGTGAGGTCCATCAAGAAGCATTTGATCCCCAGTGTAAGCGTGG
ATTGTAGTCATTAAACCTTGTTTTACGCCAAAGTTATCTTGTAAAGCTTTAGCCATTGGAGCTAAACAGTTTGTAGTACA
TGAAGCACCTGAAATAACTGTTTCAGTTCCATCAAGGATATCATGGTTAGTGTTAAATACAACTGTTTTCACGTCATCTC
CACCAGGAGCAGTGATAACAACTTTTTTAGCACCATTTTCATGTAAATGTTTTTCAGCAGCTGCTTTTTTAGCAAAGAA
ACCAGTTGCTTCAAGAACGATTTCAACACCGTCAGTTGCCCAGTCAATTTGTTCTGGATCTTTTTCAGCAGAAACTTTAA
TGAATTTTCCGTTAACGTCAAATCCACCATCTTTAACTTCTACAGTACCGTCAAAACGACCTTGAGTTTGTATCGTATTTT
AACAAGTGTGCAAGCATATTTGGGATCTGTAAAGGTCGTTGATGCGAGTAACTTCAACACCTTCCTACATTTTGAATAC
GACGG

Description	Scientific Name	Max Score	Total Score	Query Cover	E value	Per. Ident	Acc. Len	Accession
Streptococcus parauberis strain SPOF3K chromosome, complete genome	Streptococcus p...	1581	1581	100%	0.0	99.32%	2128740	CP025420.1
Streptococcus parauberis KCTC 11537, complete genome	Streptococcus p...	1576	1576	100%	0.0	99.20%	2143887	CP002471.1
Streptococcus parauberis glyceraldehyde-3-phosphate dehydrogenase gene, complete cds	Streptococcus p...	1576	1576	100%	0.0	99.20%	1011	AF421901.1
Streptococcus uberis strain SS131025-3 glyceraldehyde-3-phosphate dehydrogenase (gapC) gene, complete ...	Streptococcus u...	1266	1266	100%	0.0	92.82%	1011	KU588171.1
Streptococcus uberis strain SS131025-2 glyceraldehyde-3-phosphate dehydrogenase (gapC) gene, complete ...	Streptococcus u...	1266	1266	100%	0.0	92.82%	1011	KU588170.1
Streptococcus uberis strain SS131025-1 glyceraldehyde-3-phosphate dehydrogenase (gapC) gene, complete ...	Streptococcus u...	1266	1266	100%	0.0	92.82%	1011	KU588169.1
Streptococcus uberis 0140J complete genome	Streptococcus u...	1149	1149	100%	0.0	90.42%	1852352	AM946015.1

Isolate N. 7 (42465) - *Streptococcus ruminantium* (ID 97.42%)

TTTCGATTGTCGTACCCATGATACAACTTTCACCAATTGCTCGCCCATCCACTTCAATAACTTTTAGTTTGAGATGCGTC
AAACAATGAACAGAAATGAGATACCCACGATGTCTGAAGATACAAAGTGGATCTTCAGTGTAACCGAATGATTCAGT
AGCAGCAGCTTTCATAGCAGCGTTTACTTCTTCAGCAGTTACTTTCTTATCAAGAGTCGCAACCAATTCAGTTACAGAA
CCTGTTGGAAACTGGAACACGTTGTGCGGCACCATCAAGTTTACCATTCAATTCTGGGATTACCAAACCGATAGCTTTA
GCTGCACCAGTTGAGTTAGGAACGATGTTTGCTGCAGCAGCACGAGCACGACGAAGGTCACCACCACGGTGTGGTCC
GTCAAGAACCATTTGGTCACCAGTGTAACCGTGGATTGTAGTCATCAAACCTTTTTGAACGCCAAACGCATCGTGAAG
AGCTTTAGCCATTGGTGCCAAACAGTTTGTAGTACATGAAGCACCTGAGATAACTGTTTCAGTACCGTCAAGGATGTC
ATGGTTAGTGTTGAAAACAACTGTCTTCACATCGTTACCACCAGGAGCAGTGATAACAACTTTCTTAGCACCGTTAGCG
TGAATGTGTTGCTCAGCTTTTTCTTTAGAAGCAAAGAAACCTGTTGCTTCCAAAACAATATCTACGCCATCAGTAGCCC
AGTCAATGTTTCCTGGCTCACGCTCAGCAGAAACTTTAAACAGAATTTACCCGTTAACTTCAAAAACCACCCGTCTTTAA
CTTACAACCAGTACCCATCGAAAACGACCCTTTGA

		Score	Score	Cover	value	Ident		
Streptococcus sp. DAT741 chromosome, complete genome	Streptococcus sp. DAT741	1375	1375	99%	0.0	97.42%	2105284	CP019557.1
Streptococcus ruminantium GUT-189 DNA, complete genome	Streptococcus ruminantium	1375	1375	99%	0.0	97.42%	2081190	AP025333.1
Streptococcus ruminantium GUT-183 DNA, complete genome	Streptococcus ruminantium	1375	1375	99%	0.0	97.42%	2175328	AP025331.1
Streptococcus ruminantium GUT187T DNA, complete genome	Streptococcus ruminantium	1369	1369	99%	0.0	97.30%	2090539	AP018400.1
Streptococcus ruminantium GUT-184 DNA, complete genome	Streptococcus ruminantium	1369	1369	99%	0.0	97.30%	2115310	AP025332.1
Streptococcus suis strain NCTC10237 genome assembly, chromosome: 1	Streptococcus suis	1214	1214	99%	0.0	93.87%	2070644	LR594043.1
Streptococcus suis strain 1081 chromosome, complete genome	Streptococcus suis	1214	1214	99%	0.0	93.87%	2228089	CP017667.1
Streptococcus suis strain 0061 chromosome, complete genome	Streptococcus suis	1214	1214	99%	0.0	93.87%	2138420	CP017666.1

Isolate N. 14 (86773) - *Streptococcus parauberis* (Identity ID 98.67%)

CCTNCTCCAAGTGTCGAACAAGTGAGCAGTGTAGACATTTCATTGTCATACCATGAAACAACTTTANTAATGATTCCCA
TCAACAGTTTGTACTTTAGTTTGAGTAGCATCGAATAATGAACCGAAAGACATACCAACGATATCAGATGATACGATT
GGATCTTCAGTGTAACCATATGAATCATTAGCTGCAGCTTTCATTACTGAGTTAATTTCTTCTACTGAAGTTTCTTTATTA
AGAACTGCTACTAATTCTGTTACTGAACCTGTTGGAACTGGTACACGTTGTGCAGCACCGTCAAAGTTTACCATTTAAT
TCAGGGATAACAAGACCGATTGCTTTAGCAGCACCAGTTGAGTTAGGAACAATATTGTTAGCACCAGCACGGGCACG
ACGTAAGTCACCACCACGGTGAGGTCCATCAAGAAGCATTTGATCACCAGTGTAAGCGTGGATTGTAGTCATTAAACC
TTGTTTTACGCCAAAGTTATCTTGTAAAGCTTTAGCCATTGGAGCTAAACAGTTTGTAGTACATGAAGCACCTGAAATA
ACTGTTTCAGTTCCATCAAGGATATCATGGTTAGTGTTAAATACAACTGTTTTCACGTCATCTCCACCAGGAGCAGTGA
TAACAACTTTTTTAGCACCATTTTCATGTAAATGTTTTTCAGCAGCTGCTTTTTTAGCAAAGAAACCAGTTGCTTCAAGA
ACGATTTCAACACCGTCAGTTGCCCAGTCAATTTGTTCTGGATCTTTTTCAGCAGAAACTTTAATGAATTTTCCGTTAAC
GCCAAATCCACCATCTTTAACTTGTACAGTACCGTCA

Description	Scientific Name	Max Score	Total Score	Query Cover	E value	Per. Ident	Acc. Len	Accession
Streptococcus parauberis KCTC 11537, complete genome	Streptococcus p...	1465	1465	99%	0.0	98.79%	2143887	CP002471.1
Streptococcus parauberis strain SPOF3K chromosome, complete genome	Streptococcus p...	1459	1459	99%	0.0	98.67%	2128740	CP025420.1
Streptococcus parauberis glyceraldehyde-3-phosphate dehydrogenase gene, complete cds	Streptococcus p...	1459	1459	99%	0.0	98.67%	1011	AF421901.1
Streptococcus uberis strain SS131025-3 glyceraldehyde-3-phosphate dehydrogenase (gapC) gene, complete ...	Streptococcus u...	1177	1177	99%	0.0	92.48%	1011	KU588171.1
Streptococcus uberis strain SS131025-2 glyceraldehyde-3-phosphate dehydrogenase (gapC) gene, complete ...	Streptococcus u...	1177	1177	99%	0.0	92.48%	1011	KU588170.1
Streptococcus uberis strain SS131025-1 glyceraldehyde-3-phosphate dehydrogenase (gapC) gene, complete ...	Streptococcus u...	1177	1177	99%	0.0	92.48%	1011	KU588169.1

Isolate N. 15 (10255) - *Streptococcus uberis* (Identity ID 99.67%)

GTGCAGTGTAAGACATTTCGTTGTCATACCATGAAACAACTTTAACTAATTGATTTCCATCAACAGTTTGTACTTTAGTT
TGAGTAGCATCAAACAATGAACCGTAAGCCATACCGATGATATCAGAAGATACGATTGGGTCTTCAGTGTATCCGTAT
GAATCGTTTGCAGCTGCTTTCATTGCTGCGTTGATTTCTTCAACTGAAGTTTCTTTTTCAAGAACTGCTACTAATTCAGTT
ACTGATCCAGTTGGAACAGGAACACGTTGTGCAGCACCGTCAAGTTTACCATTTAATTCTGGGATTACAAGACCGATT
GCTTTAGCAGCACCAGTTGAGTTAGGAACAATGTTGTTTGCACCAGCACGAGCACGACGAAGGTCACCACCACGGTG
TGGTCCGTCAAGGATCATTTGGTCACCAGTGTAAGCGTGGATAGTTGTCATCAAACCTTGTTTAACACCAAAGTTATCT
TGTAAAGCTTTAGCCATTGGAGCTAAACAGTTAGTAGTACATGAAGCACCTGAAATTACAGTTTCTGTACCGTCAAGA
ATGTCATGGTTTGTGTTAAATACAACAGTTTTAACATCATCTCCACCAGGAGCTGTGATAACAACTTTTTTAGCACCGTT
AGCATGTAAATGTTTTTCAGCAGCTGCTTTTTTAGCAAAGAAACCAGTTGCTTCAAGAACGATTTCTACACCGTCAGTT
GCCCAGTCAATGTTTTCTGGATCTTTTTCAGCAGAAACTTTGATGAAGTTTCCGTTAACTTCGAATCCACCATCTTTAAC
TTCAACTGTACCGTCGAAACGACCTTGAGTTGTATCATATTTCAACAAGTGTGCAAGCATATTTGGGTCAGTAAAATCG
TTAATA

Description	Scientific Name	Max Score	Total Score	Query Cover	E value	Per. Ident	Acc. Len	Accession
Streptococcus uberis strain NCTC4674 genome assembly, chromosome: 1	Streptococcus u...	1679	1679	99%	0.0	99.78%	2024265	LS483408.1
Streptococcus uberis strain NZ01 chromosome, complete genome	Streptococcus u...	1679	1679	99%	0.0	99.78%	1863842	CP022435.1
Streptococcus uberis 0140J complete genome	Streptococcus u...	1674	1674	99%	0.0	99.67%	1852352	AM946015.1
Streptococcus uberis glyceraldehyde-3-phosphate dehydrogenase gene, complete cds	Streptococcus u...	1674	1674	99%	0.0	99.67%	1011	AF421900.1
Streptococcus uberis strain NCTC3858 genome assembly, chromosome: 1	Streptococcus u...	1668	1668	99%	0.0	99.56%	1975601	LS483397.1
Streptococcus uberis strain FSL Z2-047 glyceraldehyde-3-phosphate dehydrogenase (gapC) gene, partial cds	Streptococcus u...	1522	1522	90%	0.0	99.88%	827	GU392486.1
Streptococcus uberis strain FSL Z2-191 glyceraldehyde-3-phosphate dehydrogenase (gapC) gene, partial cds	Streptococcus u...	1517	1517	90%	0.0	99.76%	827	GU392493.1
Streptococcus uberis strain FSL Z2-262 glyceraldehyde-3-phosphate dehydrogenase (gapC) gene, partial cds	Streptococcus u...	1517	1517	90%	0.0	99.76%	827	GU392495.1
